# Transcriptomic comparison sheds new light on regulatory networks for dimorphic flower development in response to photoperiod in *Viola prionantha*

**DOI:** 10.1186/s12870-022-03732-4

**Published:** 2022-07-12

**Authors:** Qiaoxia Li, Kunpeng Li, Zhengrong Zhang, Jigang Li, Bo Wang, Zuoming Zhang, Yuanyuan Zhu, Chaochao Pan, Kun Sun, Chaoying He

**Affiliations:** 1 Life Science College, Northwest Normal University, Anning East Road 967, Anning, Lanzhou, 730070 Gansu China; 2 State Key Laboratory of Systematic and Evolutionary Botany, Institute of Botany, Chinese Academy of Sciences, Nanxincun 20, Xiangshan, Beijing, 100093 China; 3 University of Chinese Academy of Sciences, Beijing, 100049 China; 4 The Innovative Academy of Seed Design, Chinese Academy of Sciences, Beijing, China

**Keywords:** Dimorphic flower, Differentially expressed genes (DEGs), Photoperiod, Floral development, Transcriptomics, *Viola*

## Abstract

**Background:**

Chasmogamous (CH)–cleistogamous (CL) dimorphic flowers are developed in *Viola prionantha*. However, the environmental and genetic factors necessary for the CH–CL transition are unknown.

**Results:**

In the present work, short-day (SD) conditions induced CH flowers, whereas long days (LDs) triggered CL flowers in *V*. *prionantha*. Compared to fully developed CH flowers, CL flowers had less mature stamens, no nectar glands, and immature petals. Comparative transcriptomics revealed differentially expressed genes (DEGs) during CL and CH development. Core genes in the photoperiod pathway, such as *V*. *prionantha* orthologs of *GIGANTEA* (*GI*), *CONSTANS* (*CO*), and *SUPPRESSOR OF OVEREXPRESSION OF CONSTANS 1* (*SOC1*), which promote floral induction, were highly expressed in CL flowers, whereas *UNUSUAL FLORAL ORGANS* (*UFO*) and B-class MADS-box genes for floral organ identity and development showed an opposite alteration. Moreover, genes in the glycolytic process, sucrose metabolic process, and fatty acid biosynthetic process were all highly expressed in CH flowers. Interestingly, *V*. *prionantha* orthologs of the B-class MADS-box genes *APETALA3* (*AP3*) and *PISTILLATA* (*PI*) might relate to these sugar–fatty acid processes and were co-expressed with *GAIP-B-like* and *YABBY5* (*YAB5*), which regulate the development of the petal, stamen, and nectary. Compared to CH flowers, DEGs and hub genes in the most significantly correlated modules of the gene co-expression network, which are involved in abiotic and biotic responses, were upregulated in CL flowers.

**Conclusions:**

We proposed an integrative model for transcription regulation of genes in the photoperiod pathway, floral organ development, stress response, and sugar–fatty acid processes to determine CH–CL flower development in *V*. *prionantha*. Particularly, under LDs, activated *GI* may induce genes involved in the stress-response pathways, and then downregulated *AP3* and *PI* or *UFO* to inhibit the sugar–fatty acid metabolic processes, together forming CL flowers. In contrast, CH flowers were produced under SDs. This work provides novel insights into the developmental evolution of dimorphic flowers in *Viola*.

**Supplementary Information:**

The online version contains supplementary material available at 10.1186/s12870-022-03732-4.

## Introduction

Breeding systems are critical components of a population’s ability to reproduce, and these systems are often involved in the evolution of floral traits to maximize mating opportunities and fertilization success in plants [1]. Cleistogamy is a sexual system in which fertilization within individual closed flowers occurs without pollinator intervention [2]. Cleistogamy, present in 693 angiosperm species, has been classified into three categories, namely dimorphic, complete, and induced cleistogamy, with most (77.3%) being dimorphic [3]. Dimorphic cleistogamy is a specialized form of a mixed-mating system in which a single plant produces both open, potentially outcrossed chasmogamous (CH) and closed, obligately self-pollinated cleistogamous (CL) flowers. Moreover, the CL flower is a structurally modified form of the CH flower for autogamy [4, 5]; it never opens, and self-pollination occurs in a bud-like stage that is reduced compared with CH flowers. The nectar and odors of CL flowers are absent, petals are rudimentary or completely missing, stamens are often reduced in both number and size, and there are few pollen grains [6–9].

Several hypotheses have been proposed to explain natural selection leading to the maintenance of mixed-mating strategies of CH–CL flowers. In the reproductive assurance hypothesis, CL flowers are considered a backup mechanism of reproduction if CH flowers fail to set seeds, thereby extending the reproductive window of CH/CL species during the growing season [3, 10–13]. Hypothetically, to prevent inbreeding depression in dimorphic cleistogamy, CH flowers are advantageous in that they promote outcrossing and gene-flow within and among populations and produce genetically diverse progenies, thus maintaining genetic diversity, while CL increases a populations’ susceptibility to genetic drift and inbreeding depression if deleterious alleles cannot be purged [3, 11, 14–16]. The resource allocation hypothesis suggests that allocating resources to different floral morphs optimizes the use of available energy reserves [17, 18], and the production of two floral morphs is independent of each other, but one or both are correlated with a resource, typically size [19, 20] or pollinator availability [11]. CH flowers have a high energetic cost of production in some species and rely on pollinators for fertilization [3, 21]. A complex habitat model suggests that heterogeneity in the environment may lead to phenotype selection and that the frequencies of CH and CL flowers depend on environmental conditions [18]. Various environmental factors, such as photoperiod [2, 9], light intensity [8], nutrient availability [10], water supply [22], and temperature [7], influence the type of flowers produced.

The genus *Viola* (true violets) is well known for its large number of species displaying a CH/CL mixed breeding system [2, 3, 9, 16]. CH and CL flowers can exhibit different light intensities, as seen in *Viola pubescens*. As the average light quantity decreases, the odds of a CH bud developing decrease, while the odds of a CL bud increase [8]. *Viola philippica* CH flowers are induced under short-day (SD) conditions, and photoperiod extension is intended to induce CL flower formation [9, 23]. *Viola prionantha* is a perennial herb that can also develop both CH and CL flowers. However, how environmental and genetic factors affect dimorphic flower development remains unknown in this species. In this study, we observed floral development under different photoperiods in *V*. *prionantha* and compared transcriptomic variations based on full-length transcriptomes. The aims of this study were (1) to reveal the role of the photoperiod in dimorphic flower development, (2) to identify differentially expressed genes (DEGs) during CH and CL flower development, and (3) to investigate gene regulatory pathways that might determine the patterning and processes of CH and CL development. Our study contributes to the understanding of the molecular basis underlying the developmental evolution of environmentally dependent mating systems in *Viola*.

## Results

### Morphology of CH and CL flowers in *V*. *prionantha.*

**Fig. 1:**
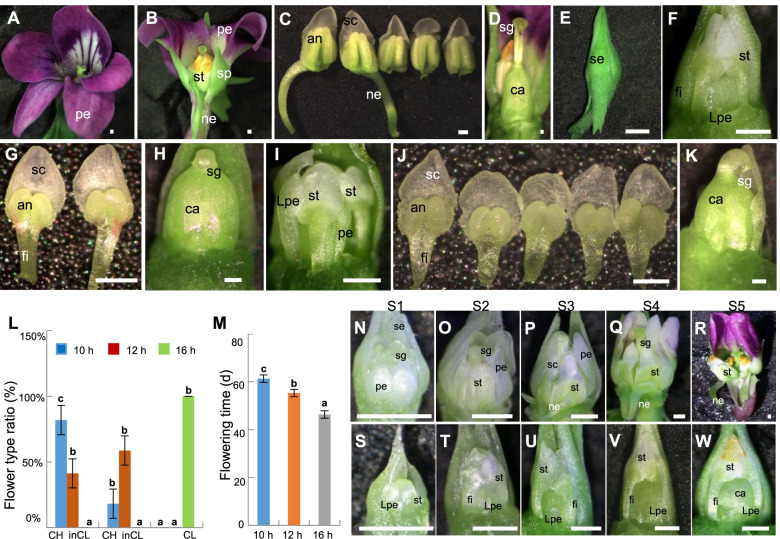
Morphological comparison between chasmogamous (CH) and cleistogamous (CL) flowers in *Viola prionantha*. (**A, B**) CH flowers. (**C**) CH flower stamens. Five stamens with invisible filaments were developed, showing distinct nectar glands. (**D**) Pistil in CH flower. (**E, F**) CL flower. (**G**) CL flower stamens. Only two stamens with visible filaments were developed. (**H**) Pistil in CL flower. The two stamens were removed. (**I**) inCL flower. (**J**) inCL flower stamens. Three to five stamens were developed with visible filaments. (**K**) Pistil in inCL flower. (**L**) The CH–CL floral transition under different photoperiods. Standard errors are provided, and lowercase letters (a, b, and c) indicate significant differences (*P* < 0.05). (**M**) Flowering time variation. (**N**–**R**) CH flowers at different developmental stages. (**S**–**W**) CL flowers at different developmental stages. Floral development as revealed by stereomicroscope analysis, and five stages (Stages 1 to 5, S1–S5 as indicated) are roughly defined. Bars = 500 µm in A–K and N–W. se, sepal; pe, petal; Lpe, lower petal; st, stamen; ca, carpel; sg, stigma; an, anther; sc, stamen cap; fi, filament; ng, nectar gland

*V*. *prionantha* is a perennial plant that can develop both CH and CL flowers at different times during the flowering season. This is quite similar to observations in *V*. *philippica* [9]. CH flowers of *V*. *prionantha* had also five large and purple petals, with the lowest petal protruding slightly at the base into a spur, and five stamens forming a cone surrounding the pistil (Fig. 1A and B). Each stamen of *V*. *prionantha* had four pollen sacs, and the lowest two stamens were attached to noticeable nectar glands (Fig. 1C). The style of the pistil was erect and exceeded the stamens (Fig. 1D). In contrast, CL flowers of *V*. *prionantha* had two stamens without nectar glands, each stamen had two pollen sacs, and the five petals were all undeveloped as primordial structures (Fig. 1E–G). The style of the pistil was curved and bent to the two big stamens (Fig. 1H). Under certain conditions, intermediate CL (inCL) flowers developed in *V*. *prionantha*, and they displayed variable characteristics. Typical ones included one to three poorly developed petals, two to five developed stamens, and a curving style of the pistil (Fig. 1I–K). Therefore, the number and size of petals and stamens in CL and inCL flowers were smaller than those of normally developed CH flowers in *V*. *prionantha*. In addition, the filaments of CL and inCL stamens were distinct compared to that of CH flowers in *V*. *prionantha*, in which they were undetectable (Fig. 1C,G and J).

### Photoperiod affects CH–CL flower development

Under natural conditions, *V*. *prionantha* developed complete CH flowers in the early spring and a mixture of inCL and CH flowers in the late spring, whereas complete CL flowers was produced in the summer and early autumn, suggesting that dimorphic flower development in this species might be regulated by the photoperiod. We thus used three photoperiods (10-, 12-, and 16-h daylight) to test this assumption in *V*. *prionantha*. The results demonstrated that complete CL flowers were formed under 16-h daylight, and both inCL and CH flowers were simultaneously developed at 10–12-h daylight in *V*. *prionantha*. Under a photoperiod of 10-h daylight, > 80% of the floral buds were developed to be CH flowers (Fig. 1L). The CH-CL formation pattern of *V*. *prionantha* in response to photoperiods is also similar to that in *V*. *philippica* [9]. In addition, flowering was induced as the photoperiod was extended in *V*. *prionantha* (Fig. 1M).

Upon naked-eye visualization of the floral buds, roughly five floral developmental stages (S1–S5) were defined in *V*. *prionantha* for both CH flowers under 10-h daylight (Fig. 1N–R) and CL flowers under 16-h daylight conditions (Fig. 1S–W). At S1, CH flowers had five obvious petals and stamens (Fig. 1N), whereas CL flowers had two stamens, and other stamens and all petals were retained as organ primordial structures (Fig. 1S). At S2, four whorl floral organs of CH flowers continued to develop, and the style of the pistil was higher than that of the stamens. The nectar glands at the base of the two stamens and spur of lower petals began to appear, while the style of the pistil in CL flowers began to bend to the two developed stamens (Fig. 1O and T). At S3, the nectar glands at the base of the two stamens were more obvious in the CH flowers, while the five petals and the other three stamens remained in the organ primordial state, and the filaments of stamen were more distinct in the CL flowers (Fig. 1P and U). At S4, the petals turned pink in the CH flowers, the filaments of the stamens became longer and finer, and the stamens were closer to the pistil in the CL flowers (Fig. 1Q and V). At S5, the petals turned purple, and the stamen cap became yellow in the CH flowers, while in the CL flowers, the stamen cap also became yellow, and the development of the three stamens and all petals was completely arrested as primordial structures (Fig. 1R and W).

To further reveal the effect of the photoperiod on CH and CL flower development, the photoperiod of *V*. *prionantha* plants was interconverted between 16- and 10-h daylight. In both cases of the “10-h to 16-h” daylight shift (Figure S1A–E) and “16-h to 10-h” daylight shift (Figure S1F–J), the CL or CH flower buds that had been induced in their original photoperiod continued to develop as their pre-programmed developmental trajectory in the new environment. Statistical analyses demonstrated that, within 8 days after the environment shift, the newly induced flower buds showed a series of inCL flower types, whereas buds initiated 8 days after the environment shift were directly transformed into CL flowers or CH flowers corresponding to the ultimate conditions of 16- or 10-h daylight (Figure S1K–P). These results indicate that the photoperiod regulates CH–CL flower development and transition in *V*. *prionantha*, and the long-day (LD) conditions can inhibit the development of stamens and petals. To understand the molecular basis for the dimorphic flower development or the CH–CL transition, we performed comparative transcriptomics between CL and CH flowers in *V*. *prionantha*.

### Transcriptome sequencing and functional annotation

**Fig. 2:**
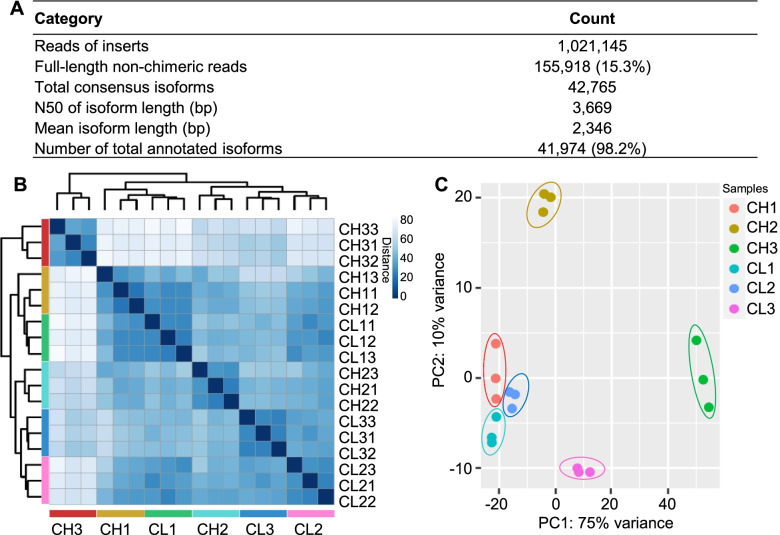
Iso-seq transcripts and hierarchical clustering of RNA-seq samples of *V*. *prionantha*. (**A**) A summary of the Iso-seq transcripts. (**B**) Clustering and heatmap analysis. The sample distances are represented by variations from dark blue (high similarity) to light blue (low similarity). (**C**) PCA for all 18 samples based on the expression values of transcriptome-wide expression profiles

We first sequenced the full-length transcriptome of *V*. *prionantha* of whole plants, including the roots, stems, leaves, and involved floral buds, using the Pacific Biosciences Sequel platform, and generated 1,021,145 reads in total (Fig. 2A, Table S1). There were 155,918 full-length non-chimeric and clean reads, and 42,765 consensus isoforms were identified (Fig. 2A). The mean and N50 of the isoform length were 2346 bp and 3669 bp, respectively (Fig. 2A, Figure S2A). Functional annotations of the longest open reading frame (ORF), as the coding sequence (CDS), were performed using BLAST, Blast2GO, and InterProScan5, and 98.15% of transcripts were annotated (Fig. 2A, Tables S2 and S3). The NR database had the highest annotation rate, accounting for 96.1% of all transcripts, and the GO database had the lowest annotation rate, accounting for 35.39% of all transcripts (Table S3). The total number of transcripts annotated by all seven databases was 17,102, accounting for 40.74% of all transcripts (Figure S2B). With NR annotation, approximately 74% of all transcripts of *V*. *prionantha* were similar to *Jatropha curcas*, *Populus trichocarpa*, *P*. *euphratica*, and *Ricinus communis* (Figure S2C). To reveal the evolutionary relationship of *V*. *prionantha*, we first used Orthofinder to identify orthologous genes in the amino acid sequences of 17 other plant species, including *P*. *deltoids*, *P*. *trichocarpa*, and *Arabidopsis thaliana*, and a total of 24,585 gene orthogroups were constructed (Table S4). Phylogenetic analysis with these orthologous sequences using *H*. *vulgare* and *O*. *sativa* as an outgroup supported the notion that *V*. *prionantha* was the closest relative to *P*. *deltoides* and *P*. *trichocarpa* (Figure S3). We then evaluated the expansion and contraction of certain gene families and found that a total of 2555 families were expanded (73 significant, *P* ≤ 0.05), while 4586 gene families were contracted (120 significant, *P* ≤ 0.05) in *V*. *prionantha* (Figure S3). GO and KEGG enrichment analyses of the 1405 genes in the significantly expanded gene families (Table S5) revealed GO (Figure S4A, Table S6) and KEGG (Figure S4B, Table S6) categories/pathways, including ethylene-responsive proteins, lipoxygenase, and jasmonate ZIM domain protein, that might be involved in stress response and flower development.

### RNA-seq between CH and CL flowers

To observe general variation patterns at the transcriptomic level between CH and CL flowers, three developmental stages (CH1-3 or CL1-3) of each flower type, corresponding to S1, S3, and S5, were subjected to RNA-seq analysis. We sequenced the 18 samples using the BGISEQ-500 platform and generated approximately 23.98 Mb RNA clean reads per sample on average (Table S7). By mapping them to the identified unique isoforms, 67.62% of the RNA clean reads were reasonably aligned (Table S8). Moreover, most transcripts were completely covered with uniform read coverage alone in the body using the RNA-seq data (Figure S5A and B). Gene expression correlation with samples showed that the samples in the three biological repeats at the same developmental stage had a high correlation, whereas the samples in different developmental stages had a relatively low correlation (Fig. 2B). Principal component analysis (PCA) also showed that three repeated samples at the same developmental stage were clustered together, whereas the samples at different stages were far away from each other (Fig. 2C). Thus, all generated RNA-seq data could be used for further analysis.

### GO enrichment of stage-specific DEGs between CH and CL flowers

To identify genes related to dimorphic floral development in *V*. *prionantha*, we performed a DEG analysis by comparing the CH and CL flowers for the developmental stages (S1, S3, and S5) with DESeq2. A total of 162, 673, and 1716 genes were respectively found to be differentially expressed in S1, S3, and S5 (Table S9), and a Venn diagram revealed the distribution and relationship of these DEGs among these three developmental stages (Figure S6A). To further reveal the contribution of the DEGs’ variation to the CH-CL floral transition, we investigated the potential biological functions of the stage-specific DEGs through GO enrichment analysis.

**Fig. 3:**
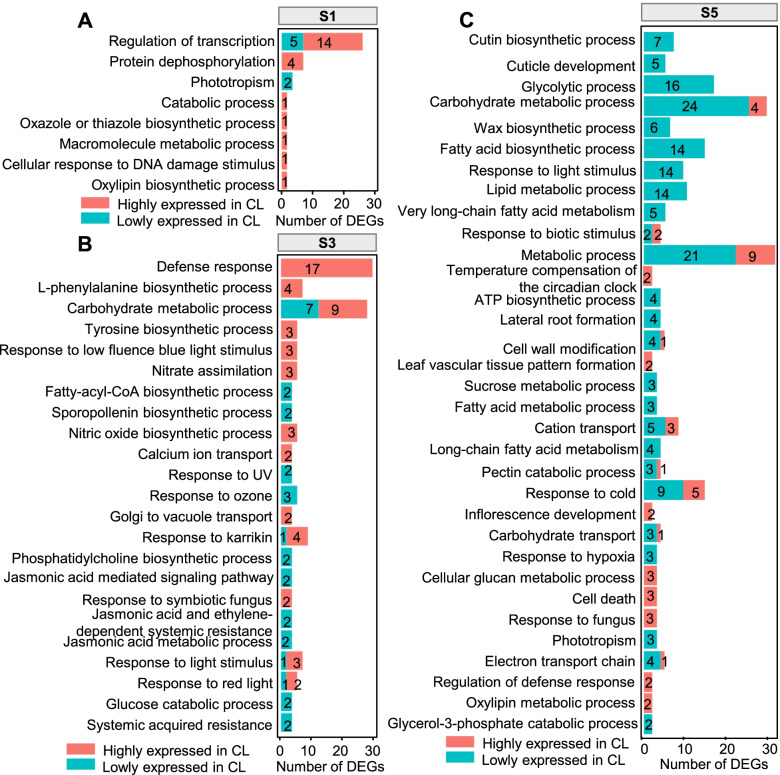
GO enrichment analyses of DEGs during CH and CL development in *V*. *prionantha. *(**A**) GO terms significantly enriched in Stage 1 (S1). (**B**) GO terms significantly enriched in S3. (**C**) GO terms significantly enriched in S5. *p*-value < 0.02. Red bar represents high expression of DEGs in CL, and blue represents low expression of DEGs in CL

In S1, eight enriched GO terms were constructed with 162 DEGs between the CH and CL flowers, and the most dominant was transcription regulation (Fig. 3A, Figure S6A, Table S10). Of these, 5 genes were downregulated and 14 genes were upregulated in CL flowers relative to CH flowers. The genes downregulated in CL flowers included four B-class MADS-box genes *PISTILLATA* (*PI*, *Vp42765*), *APETALA3* (*AP3*, *Vp31710*, *Vp32333*, *Vp33366*), and one F-box transcription factor (TF) gene *UNUSUAL FLORAL ORGANS* (*UFO*, *Vp21783*) (Figure S6B), which might be critical for promoting the initiation and development of petals and stamens (Table S11). While the 14 upregulated TF genes in CL flowers included *SUPPRESSOR OF OVEREXPRESSION OF CONSTANS 1* (*SOC1*, *Vp24825*), *FLAVIN-BINDING KELCH REPEATF-BOX1/ ADAGIO3* (*FKF1*/*ADO3*) (*Vp13028*), *ETHYLENE RESPONSE FACTOR53* (*ERF05*3) (*Vp15804*, *Vp19048*), and *ERF4* (*Vp23464*) (Figure S6B), which might be related to the circadian clock, carbohydrate synthesis, and defense response (Table S11).

A total of 23 enriched GO terms were identified for the 571 DEGs specific in S3 relative to S1, which mainly included defense response, carbohydrate metabolic process, nitrate assimilation, sporopollenin biosynthetic process, calcium ion transport, fatty-acyl-CoA biosynthetic process, glucose catabolic process, and jasmonic acid-mediated signaling pathways (Fig. 3B, Figure S6A, Table S10). Notably, 17 DEGs were involved in the defense response, and they were all upregulated in CL flowers compared with CH flowers (Fig. 3B). Of these, 16 DEGs involved in the carbohydrate metabolic process included 7 downregulated genes and 9 upregulated genes in CL flowers (Fig. 3B). In addition, genes in sporopollenin biosynthetic, fatty-acyl-CoA biosynthetic, and glucose catabolic processes were downregulated in CL flowers compared with CH flowers, and genes in nitrate assimilation and calcium ion transport were upregulated in CL flowers (Fig. 3B).

A total of 33 enriched GO terms were identified for the 1331 specific DEGs in S5 and included genes in the glycolytic process, carbohydrate metabolic process, and fatty acid biosynthetic process (Fig. 3C, Figure S6A, Table S10). Most of these genes were downregulated in CL flowers compared with CH flowers, whereas the genes involved in temperature compensation of the circadian clock were upregulated in CL flowers compared with CH flowers (Fig. 3C).

Taken together, the number of DEGs increased as the flower developed. The differentially expressed TF genes occurred at the earlier stages, and then the genes involved in metabolic processes related to sugar and fatty acids became differentially expressed between CH and CL flowers at late developmental stages, implying the essential role of these DEGs and their programmed regulation in the divergence of CH and CL flowers in *V*. *prionantha*.

### Expression patterns of MADS-box genes in *V*.* prionantha.*

**Fig. 4:**
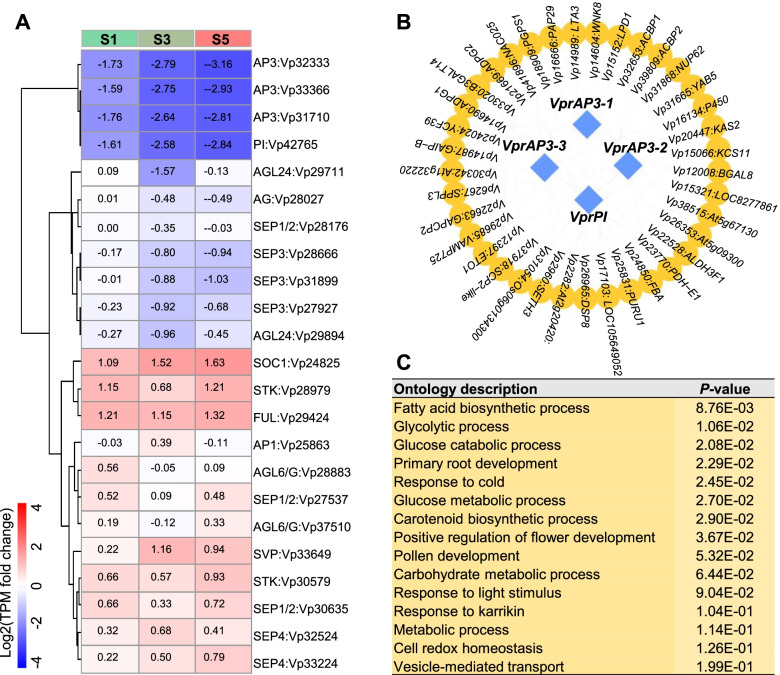
Expression of MADS-box genes and *VprAP3*/*VprPI* co-expression networks in *V*. *prionantha*. (**A**) Heatmap displaying the expression profile of the identified MADS-box genes at three developmental stages. Red represents high gene expression in CL, and blue represents low expression in CL. The fold change of differential expression (CL/CH) during the three stages (S1, S3, and S5) is labeled on the top. (**B**) *VprAP3* and *VprPI* co-expression network analysis. All co-expressed genes (Mutual-Rank < 10) with *VprAP3* and *VprPI* are indicated by yellow circles, and *VprAP3* and *VprPI* are highlighted by blue diamonds. (**C**) Significantly enriched GO terms of the 37 co-expressed genes of *VprAP3* and *VprPI*

Downregulation of B-class MADS-box genes induces CL flowers in *V*. *philippica* [9]. The downregulated genes enriched in the regulation of transcription were mainly B-class MADS-box genes. We therefore characterized this gene family and obtained 23 MIKC-type MADS-box genes from the whole transcript data of *V*. *prionantha*. Phylogenetic analysis showed that the MADS-box genes of *V*. *prionantha* always clustered with their homologous genes in *A*. *thaliana* (Figure S7), suggesting their orthology. An expression profile of the identified MADS-box genes in three developmental stages of CH and CL flowers in *V*. *prionantha* showed that a high level of expression of the four B-class MADS-box genes *VprAP3* (*Vp31710*, *Vp32333*, *Vp33366*) and *VprPI* (*Vp42765*) was observed in CH flowers relative to CL flowers (Fig. 4A, Table S12). In addition, the level of expression of the C-class MADS-box gene *AGAMOUS* (*AG*, *Vp28027*) was also higher in the CH flowers. However, the photoperiod-induced genes, such as *SOC1* (*Vp24825*) and *FRUITFULL* (*FUL*, *Vp29424*), D-class MADS-box gene *SEEDSTICK* (*STK*, *Vp30579*), and *AGAMOUS-LIKE6* (*AGL6*, *Vp28883*, *Vp37510*), showed lower expression levels in CH flowers than in CL flowers (Fig. 4A, Table S12).

We focused particularly on genes co-expressed with *VprAP3/VprPI*. A total of 37 genes showed the highest correlation (PCC > 0.9) with *VprAP3/VprPI* expression (Fig. 4B, Table S13). GO enrichment analysis showed that these genes were mainly involved in the fatty acid biosynthetic process, glycolytic process, carbohydrate metabolic process, glucose metabolic and catabolic process, positive regulation of flower development, and pollen development (Fig. 4C, Tables S14 and S15). Moreover, the expression of most genes co-expressed with *VprAP3* and *VprPI* was higher in CH flowers of the three developmental stages than in CL flowers (Figure S8). The results suggested that glycolytic, fatty acid biosynthetic, and carbohydrate metabolic processes were putatively controlled by *VprAP3/VprPI* for dimorphic flower development. In addition, *VprAP3/VprPI* were also co-expressed with *GAIP-B-like* (*Vp14987*) and *YABBY5* (*YAB5*, *Vp31665*) genes that regulate the development of petals, stamens, and nectaries (Fig. 4C, Table S15).

### Expression profile of genes related to sugar–fatty acid processes in *V*.* prionantha.*

**Fig. 5:**
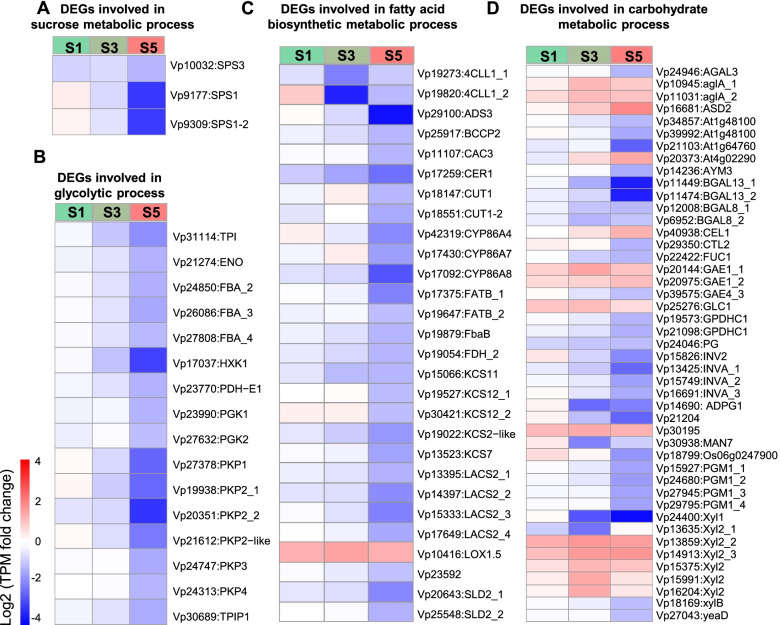
Expression profiles of the DEGs related to sugar–fatty metabolisms in *V*. *prionantha. *(**A**) Sucrose metabolic processes. (**B**) Glycolytic process metabolic processes. (**C**) Fatty acid biosynthetic metabolic processes. (**D**) Carbohydrate metabolic processes. In heatmaps, red represents highly expressed DEGs in CL, and blue represents lowly expressed DEGs in CL

Genes involved in sucrose metabolic, glycolytic, and fatty acid biosynthetic processes were highly expressed in CH flowers compared to CL flowers, especially at S5 (Fig. 3C, Table S10). A higher expression level of sucrose metabolic process genes, i.e., *SUCROSE-PHOSPHATE SYNTHASE 1* (*SPS1*, *Vp9177*, *Vp9309*) and *SPS3* (*Vp10032*), which encode the key enzymes for sucrose synthesis, was observed in CH flowers than in CL flowers (Fig. 5A, Table S16). In the GO term of the glycolytic process, 16 genes were significantly differentially expressed, and most of them were upregulated in CH flowers at three developmental stages compared to CL flowers (Fig. 5B, Table S16).

Twenty-eight genes in the fatty acid biosynthetic process were significantly differentially expressed between the CH and CL flowers (Fig. 5C). The expression level of genes encoding biotin carboxyl carrier protein 2 (*BCCP2*, *Vp25917*), ketoacyl-CoA synthase (*KCS*, *Vp15066*, *Vp19527*, *Vp30421*, *Vp19022*, and *Vp13523*), and long-chain acyl-CoA synthetase (*LACS*, *Vp13395*, *Vp14397*, *Vp15333*, and *Vp17649*) participating in fatty acid biosynthesis was stably higher in CH flowers than in CL flowers during development, while *LINOLEATE 9S-LIPOXYGENASE 5* (*LOX1*.*5*, *Vp10416*) was less expressed in CH flowers than in CL flowers (Fig. 5C, Table S16). Moreover, the expression of genes involved in lignin and cutin synthesis, such as *COUMARATE—COA LIGASE* (*4CLL1*, *Vp19273*, and *Vp19820*), was also constitutively higher in CH flowers than in CL flowers during development (Fig. 5C, Table S16). These results suggest that CH floral development might require more fatty acids, which could promote stamen fertility, increase the osmotic pressure of stamens and petals, and absorb and transport metal ions in floral organs (Table S16).

Additionally, there were 45 DEGs in the carbohydrate metabolic process, of which 14 genes were downregulated and 31 genes were upregulated in CH flowers compared to CL flowers (Fig. 5D). Most gene-related glycosidase families, including xylosidase (*Xyl*, *Vp13859*, *Vp14913*, *Vp15375*, *Vp15991*, and *Vp16204*), Glucan endo-1,3-beta-glucosidase (*GLC1*, *Vp25276*), and cellulose1 (*CEL1*, *Vp40938*), were stably downregulated in CH flowers compared to CL flowers, whereas polygalacturonase (PG) genes, including *Vp34857*, *Vp39992*, *Vp24046*, and *Vp14690*, were upregulated in CH flowers (Fig. 5D, Table S16).

### Gene co-expression networks during CH–CL flower development

**Fig. 6:**
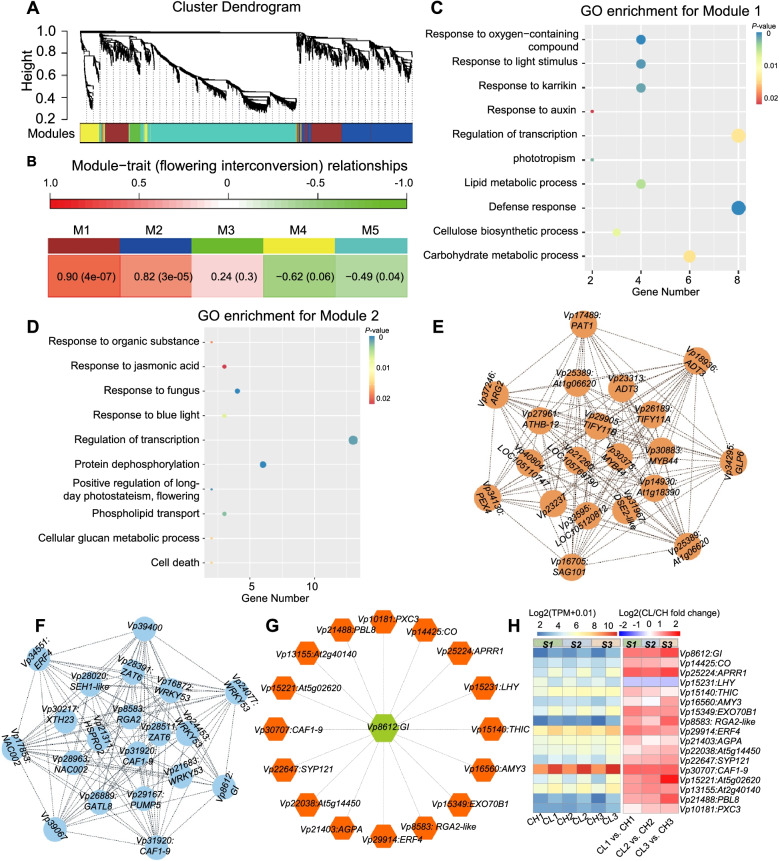
WGCNA during the interconversion between CH and CL flowers of *V*. *prionantha. *(**A**) Dendrograms of all DEGs are clustered based on a dissimilarity measure (1-TOM). All leaves in the tree represent a single gene, and the major tree branches constitute five distinct modules (M1–M5) shown in different colors. (**B**) Heatmap of the module–trait relationships. The trait refers to the structural changes during interconversion between chasmogamous and cleistogamous flowers. The top color panel represents Pearson’s correlation coefficient, with red for positive correlation and blue for negative correlation. Each cell contains the corresponding correlation and *p*-value. (**C**, **D**) GO enrichment analysis of module1 (C) and module2 (D). In each analysis, the top 10 GO terms are shown. *P*-values represent the significance of enrichment. Circles indicate the target genes, and the sizes are proportional to the number of DEGs. (**E**, **F**) The top 20 genes ranked by the Maximal Clique Centrality (MCC) score identified in module1 (E) and module2 (F). Nodes represent hub genes and are labeled on circles, and co-expressed genes are linked by dashed gray lines. (**G**) *GIGANTEA* (*Vp8612:GI*) gene co-expression network analysis. All co-expression genes (Mutual-Rank < 10) are labeled in red hexagons. (**H**) Expression profile of the co-expressed genes with *Vp8612:GI*. Heatmap displays the expression in transcripts per million (TPM) (left) and fold change (CL/CH) (right) in each floral stage

To facilitate the understanding of the regulatory network of flowering interconversion, we built weighted gene co-expression networks with all DEGs (2064 genes) among the three different developmental stages of *V*. *prionantha*. We chose a power of *β* = 10 based on the scale-free topology criterion to generate a hierarchical clustering tree (Figure S9A and B). A total of five co-expression modules (mergeCutHeight = 0.2) M1–M5 marked with different colors were ultimately identified, and they had a gene number in a module ranging from 71 to 969 (Fig. 6A). Correlations between the modules’ eigengenes (MEs) and genotypes/stages showed that all modules were obviously correlated with flower phenotypes, and M1 (383 genes) and M2 (478 genes) modules exhibited the most significant correlation (Fig. 6B). Significant correlations were also detected between stage-specific gene expression and module membership (MM) in each module (Figure S10), particularly in M1 and M2 modules (Figure S10A and B). Therefore, genes in M1 and M2 might play a role in CH–CL flower development. GO annotations revealed that most genes in M1 were involved in defense response, cellulose biosynthetic process, response to oxygen-containing compounds, response to karrikin, regulation of transcription, lipid metabolic process, and carbohydrate metabolic process (Fig. 6C, Table S17). The top 10 enriched GO terms in M2 were related to the response to jasmonic acid, response to fungus, response to blue light, regulation of transcription, protein dephosphorylation, positive regulation of LD photoperiodism, and flowering (Fig. 6D, Table S18).

To further identify the key genes essential for CH–CL flower development, we extracted hub genes in the M1 and M2 modules. The top 20 genes with the highest maximum clique centrality (MCC) values were considered hub genes (Fig. 6E and F, Tables S19 and S20). For instance, genes in M1, including *HOMEOBOX-LEUCINE ZIPPER PROTEIN 12* (*ATHB-12*), *PHYTOCHROME A SIGNAL TRANSDUCTION 1* (*PAT1*), *MYB44*, *SENESCENCE-ASSOCIATED GENE 101* (*SAG101*), and *GERMIN-LIKE PROTEIN 6* (*GLP6*), were responsible for abiotic and biotic stress in other plant species (Fig. 6E, Table S21), while genes in M2, including *ERF4* and *XYLOGLUCAN ENDOTRANSGLUCOSYLASE/HYDROLASE 23* (*XTH23*), were also related to stress responses (Fig. 6F, Table S21). Notably, a hub gene *Vp8612* encoding *V*. *prionantha* GIGANTEA (VprGI) was found in M2, and this gene belonged to the GO terms of positive regulation of LD photoperiodism flowering (Fig. 6F, Table S21). These genes were expressed at a low level in CH flowers compared with CL flowers (Figure S11A and B). We then focused particularly on the *VprGI* co-expression network in *V*. *prionantha*. The expression of 17 genes showed the highest correlation (PCC > 0.8, MR ≤ 10) with *VprGI* expression (Fig. 6G, Table S22), and they included *V*. *prionantha CONSTANS* (*VprCO*), *PSEUDO-TYPE RESPONSE REGULATORS1* (*APRR1*), *LATE ELONGATED HYPOCOTYL* (*LHY*), *EXOCYST COMPLEX COMPONENT 70B1* (*EXO70B1*), *ERF4*, *PBS1-like kinases 8* (*PBL8*), and *PXY-correlated* (*PXC3*), which are involved in the circadian clock and defense response progress (Table S23). The expression of these genes, except for *LHY*, was at low levels in CH flowers relative to CL flowers (Fig. 6H). In addition, *PHOSPHOMETHYLPYRIMIDINE SYNTHASE* (*THIC*), which plays an important role in linking the circadian clock and metabolic rhythm, was also at low levels in CH flowers relative to CL flowers in *V*. *prionantha* (Table S23).

The results suggest that LD light could induce the co-expression of floral inducers, such as *VprGI* and *VprCO*, and coincidently stress-response genes during the induction of CL flowers in *V*. *prionantha*.

### Expression of key TF genes correlated with CH–CL development

**Fig. 7:**
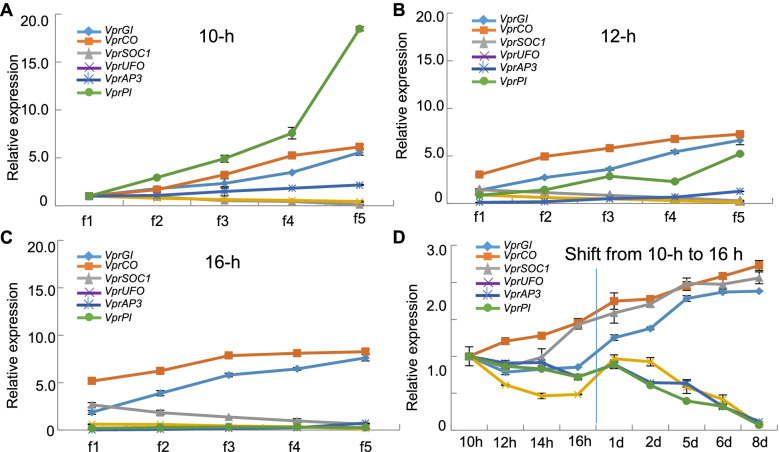
The expression of six candidate genes during CH–CL flower development in *V*. *prionantha*. (**A**) Expression of *VprGI*, *VprCO*, *VprSOC1*, *VprUFO*, *VprAP3*, and *VprPI* under 10-h daylight. (**B**) Expression of the six genes under 12-h daylight. (**C**) Expression of the six genes under 16-h daylight. (**D**) Expression variation of the six genes in response to different times of illumination and days after switching from 10-h daylight to 16-h daylight. Gene expression in flower buds around S1 (f1), S2 (f2), S3 (f3), S4 (f4), and S5 (f5) under 10-, 12-, and 16-h daylight (A–C) or equal mixture of f1 and f2 buds under 10, 12, 14, and 16 h of illumination, and 1, 2, 5, 6, and 8 days after the plants switched from 10-h daylight to 16-h daylight (D) evaluated using qRT-PCR. The gene expression in f1 under 10-h daylight was set to 1 in A, B, and C, while gene expression in 10-h illumination was set to 1 in D. Three independent biological samples were used in the analyses. Means and standard errors are presented. Significant differences are presented in Table S24

Six key candidate regulatory genes (*GI*:*Vp8612*, *CO*:*Vp14425*, *SOC1*:*Vp24825*, *UFO*:*Vp21783*, *AP3*:*Vp33366*, and *PI*:*Vp42765*) in *V*. *prionantha* that might be essential for CH–CL floral transition were targeted and designated *VprGI*, *VprCO*, *VprSOC1*, *VprUFO*, *VprAP3*, and *VprPI*, respectively. Their expression variations during CH and CL development were validated using qRT-PCR. The expression of *VprGI*, *VprCO*, and *VprSOC1* was at high levels in CL flowers under 16-h daylight relative to those subjected to 12- and 10-h daylight, whereas the expression of *VprUFO*, *VprAP3*, and *VprPI* was at high levels in CH flowers under 10-h daylight relative to inCL and CL flowers under 12- and 16-h daylight (Fig. 7A–C, Table S24). These results were consistent overall with the transcriptome data. We also investigated the expression of these genes in *V*. *prionantha* plants under 10-h daylight after they were switched to 16-h daylight conditions. The expression of *VprGI*, *VprCO*, and *VprSOC1* in the first and second stages of floral buds were elevated with continuous increasing illumination and became significantly upregulated after a one-day shift from 10- to 16-h daylight, while expression variation of *VprUFO*, *VprAP3*, and *VprPI* showed the opposite trend (Fig. 7D, Table S24).

## Discussion

CH flowers possess colored petals and nectar to attract pollinators, while CL flowers are obligate self-pollinators with vestigial or undeveloped petals, and no nectar. The petal and stamen primordia were initiated, and further development was arrested during CL floral development. No morphological differences at floral induction or floral organogenesis were observed between CH and CL flowers in *V*. *prionantha*, and flower morphological differentiation in *V*. *prionantha* of the two flower types should be determined after floral organogenesis. This is similar to observations in *V*. *philippica* [9]. In response to the photoperiod, GAs interact with B-class homeotic genes and regulate dimorphic flower development in *V*. *philippica* [23]. In the present work, the photoperiod played a role in CH–CL development. Although no differential expression of GA-biosynthetic genes was found (this might be due to the sampling strategy), we revealed several novel findings at transcriptomic levels. We proposed an integrative role for transcription regulation of genes involved in photoperiod–circadian pathways, organ development, stress response, and sugar–fatty acid metabolic processes in response to photoperiod for CH–CL development, shedding new light on the understanding of the molecular basis underlying the developmental evolution of CH–CL transition in *V*. *prionantha*.

### Photoperiod regulates dimorphic flower development in *V*.* prionantha*

Many environmental factors can affect CH–CL development [8, 9, 22]. The CH/CL system is favored in a heterogeneous parental environment if individuals are exposed to a selection regime in which each flower type is favored in a particular environment [18]. The photoperiod can influence the timing of the floral transition, which leads to the conversion of the shoot apical meristem into a floral meristem and subsequently to the formation of flowers in plants [24, 25]. Our study showed that photoperiod also affected dimorphic flower development, and the phenology of the two flower types in *V*. *prionantha* was mainly determined by photoperiod. SD light induced CH flower development, whereas LD light induced CL flower development. This phenomenon was also observed in other *Viola* species, such as *V*. *odorata* and *V*. *philippica* [2, 9]. Moreover, when the *V*. *prionantha* plants with CL flowers under LD light or CH flowers under SD light were interconverted, the newly induced flowers showed a series of inCL flower types, and finally transformed into CH flowers or CL flowers. The transition depended on the duration of the photoperiod in the new environment in a manner of “the longer, the more thorough.” Therefore, the photoperiod regulates dimorphic flower formation in *V*. *prionantha*.

### Transcription regulation of CH and CL development in *V*. *prionantha*

Gene expression variation in response to photoperiod variation is the major molecular basis underlying the formation of CH or CL flowers. Driven by this presumption, we comparatively investigated the transcriptomic variations. In line with the morphological variation pattern, relatively fewer genes were identified as DEGs in the earlier developmental stages, while more genes became DEGs as the developmental course advanced. Moreover, transcription regulation was different in the early stages, while the genes involved in metabolic processes diverged in the later developmental stages. Similarly, the DEGs in stage 1 were mainly involved in the regulation of transcription. B-class MADS-box genes *VprAP3* and *VprPI* and one F-box TF gene *VprUFO* were obviously upregulated in CH flowers compared with CL flowers in *V*. *prionantha*.

The floral ABC model explains how three major function class genes (A-, B-, and C-class) specify the identity of the four floral organ types. A function specifies sepal identity [26, 27], while A and B activities together specify petal identity [26–29]. B plus C activity specifies stamens [30], while C activity specifies carpel identity [31]. A pair of MADS-box genes, *AP3* and *PI*, in *Arabidopsis* encode B-function activity [32, 33]. B-class MADS-box genes are involved not only in the specification of organ identity of petals and stamens but also in the control of organ maturation [9, 34, 35]. In *V*. *philippica*, the downregulation of B-class MADS-box genes during the late developmental stages may result in organ size reduction and developmental abortion, and the expression of these genes is induced by SDs and inhibited by LDs [9]. A similar expression variation of B-class genes during CH–CL development was observed in *V*. *prionantha*. Therefore, LDs could also inhibit the development of petals and stamens by directly or indirectly inhibiting the expression of *VprAP3* and *VprPI* genes, thus producing CL flowers in *V*. *prionantha*. In particular, *GAIP-B-like* and *YAB5* were co-expressed with *VprAP3* and *VprPI*, and they were upregulated in CH flowers compared with CL flowers in *V*. *prionantha*. *GAIP-B-like* encodes the DELLA protein, regulates the expression of *AP3*, and is involved in petal and stamen development [36]. *YAB5* is indispensable for pseudonectary development [37]. These results further support the transcription regulation of B-class MADS-box genes in CH–CL development.

*UFO* is not a component of the floral ABC model. However, it is required to initiate the floral program [38]. *UFO* is expressed in the early development of petals and stamens in *Arabidopsis*, probably restricting the B-class MADS-box genes’ expression domain to these organs [39, 40]. Moreover, *UFO* expression is also required for normal petal blade outgrowth after petal identity has been established [41]. Furthermore, UFO is involved in activating *CRABS CLAW* (*CRC*) during nectary and carpel development [42]. We showed that *VprUFO* was significantly upregulated in CH flowers compared with CL flowers, especially at stage 1, and induced by SD light. Therefore, the photoperiod may, directly or indirectly, regulate *VprUFO*, which is further involved in influencing *VprAP3* and *VprPI*, thus controlling petal and stamen development of either CH or CL flowers in *V*. *prionantha*.

### Role of genes in sugar–fatty acid metabolic processes in dimorphic flower development

Sugar and fatty acids play important roles in floral development and function, for example, flower opening [43, 44]. The expression of most genes in the glycolytic, fatty acid biosynthetic, and sucrose metabolic processes was higher in CH flowers than in CL flowers, especially at the third developmental stage. Moreover, genes co-expressed with *VprAP3* and *VprPI* involved in the glycolytic, fatty acid biosynthetic, and sucrose metabolic processes were stably higher in CH flowers than in CL flowers throughout development. Therefore, B-class MADS-box genes might be involved in regulating sugar–fatty acid processes, thus playing a role in determining CH or CL under different photoperiods. However, evidence of the regulation of sugar–fatty acid metabolism by B-class MADS-box genes has not yet been reported, which requires investigation.

It is worth mentioning that *PG* and *Xyl* not only participate in the carbohydrate metabolic process but are also involved in flower development. Overexpressing *PG* displays higher proportions of flowers with extra petals, suggesting *PG* involvement in floral organ patterning [45], while the knockout of *Arabidopsis PG* genes results in the generation of tetrad pollen [46]. The expression of *PG* (*Vp34857*, *Vp39992*, *Vp24046*, and *Vp14690*) was low in CL flowers and higher in CH flowers. Xyl acts on xyloglucans in the cell wall to promote cell expansion, which might involve the elongation of anther filaments and the degradation of anthocyanin [47, 48]. In the present study, a high level of expression of *Xyl2* (*Vp13859*, *Vp14913*, *Vp15375*, *Vp1599*, and *Vp16204*) was observed in CL flowers, with a low level in CH flowers. The results suggested that *Xyl2* might be involved in the elongation of anther filaments and the degradation of anthocyanin in CL flowers of *V*. *prionantha*.

Our results showed a correlated variation between sugar–fatty acid metabolic gene expression and flower types, implying the role of sugar–fatty acid metabolism in dimorphic flower development.

### Gene co-expression of the photoperiod pathway and defensive responses

Many genes in the photoperiod pathway related to the circadian clock are involved in flower induction, such as *GI*, *FKF1/ADO3*, *CO*, *FT* (*FLOWERING LOCUS T*), *FUL*, and *SOC1* in *Arabidopsis*. The expression profile of *GI* is controlled by the circadian clock, and the GI protein is required for the function of *FKF1* in regulating *CO* transcription [49–51]. CO plays a central role in activating *FT* expression for the induction of *SOC1*. *SOC1* promotes floral transition [52, 53], which is, in part, activated by the floral identity gene *LEAFY* (*LFY*) [54–56]. Furthermore, *FUL* has been shown to promote flowering in a partially redundant manner with *SOC1* [57]. In *V*. *prionantha*, the expression of genes controlled by the circadian clock was at a high level in CL flowers relative to CH flowers. The study showed that LDs induced the *V*. *prionantha* to bloom earlier, probably because LD promoted the expression of the orthologous genes of *GI*, *FKF1/ADO3*, *CO*, *FUL*, and *SOC1* in *V*. *prionantha*.

The present study showed that 17 DEGs in S3 involved in the defense response were upregulated in CL flowers compared with CH flowers. Moreover, the *LOX* (*lipoxygenase*) gene family in *V*. *prionantha* was significantly expanded, and the level of expression of *LOX1*.*5* was lower in CH flowers than in CL flowers. LOX, which are precursor molecules of JA biosynthesis, are involved in stress responses and filament development in flowers [58, 59]. Furthermore, most of the hub genes in module1 and module2 of WGCNA responded to abiotic and biotic stress, such as *ATHB-12*, *MYB44*, *PAT1*, *SAG101*, *GLP6*, *ERF4*, and *XTH23*, and the level of expression of these genes was higher in CL flowers than in CH flowers at three development stages.

Interestingly, except for circadian clock genes, genes in the *VprGI* co-expression network were resistant to stress, such as *EXO70B1*, *ERF4*, *PBL8*, and *PXC3*, and these genes were expressed at a high level in CL flowers compared with CH flowers. Moreover, the gene family of the response to ethylene in *V*. *prionantha* was also significantly expanded, and ERF4 belongs to the ethylene response factor [60]. Co-expression of *VprGI* and defense response genes indicates that these stress-response genes might be controlled by *VprGI* or other circadian clock genes in *V*. *prionantha*. The circadian clock genes respond to various abiotic and biotic stressors in *A*. *thaliana* and crops [61], and *Arabidopsis GI* is involved in signaling pathways for various abiotic stressors [62, 63]. For example, the EEL (ENHANCED EM LEVEL)-GI complex positively regulates diurnal ABA synthesis by affecting the expression of *NCED3* (*9-CIS-EPOXYCAROTENOID DIOXYGENASE 3*) and contributes to drought tolerance in *Arabidopsis* [63]. In addition, the GI-mediated regulation of *CDFs* (*CYCLING DOF FACTOR*) contributes to the response to freezing temperatures [62]. Therefore, circadian clock stress-response gene modules might be involved in CH–CL development in *V*. *prionantha*.

### A novel integrative model for CH–CL development

**Fig. 8:**
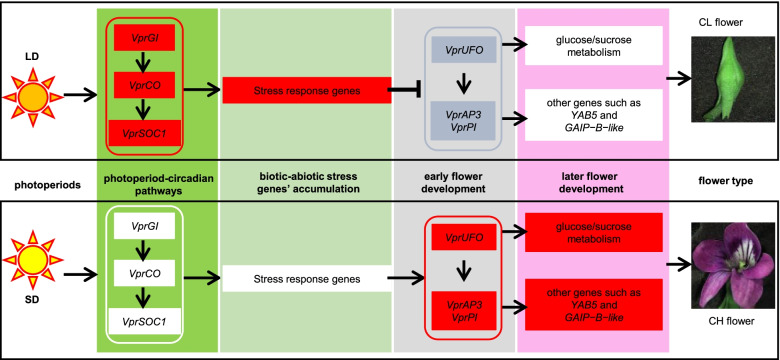
An integrative regulatory model of CH and CL development in *V*. *prionantha. *In the gene module, no gene expression variation, upregulation, and downregulation are shown in white, red, and blue, respectively. LD, long day; SD, short day. ↓indicates activation, and ⊥indicates repression

In *Arabidopsis*, LD light induces flowering induction genes, such as *GI*, *FKF1/ADO3*, *FT*, *CO*, *SOC1*, and *AGL24*, and these proteins in turn promote the expression of flower meristem genes *LFY* and *AP1* [52]. Once the floral meristem has been specified, AP1 and LFY activate floral organ identity genes [64], LFY participates with UFO in the regulation of *AP1* and *AP3* transcription [39, 40], and WUS co-regulates the expression of *AG* [65, 66]. However, in *V*. *prionantha*, LDs also induced the expression of *VprGI*, *VprFKF1/ADO3*, *VprCO*, and *VprSOC1*, but the expression of *VprAP1*, *VprAP3*, and *VprAG* was not promoted but repressed, thus producing CL flowers. The opposite variation was observed under SD, thus producing CH flowers. Coincidently, the co-expression of circadian clock-related genes and stress-response genes in the photoperiod pathway indicated that circadian clock genes could promote stress-response genes under LDs, which could inhibit the expression of genes related to floral organ development, especially *VprUFO*, *VprAP3*, and *VprPI*. Similarly, *cis*-elements of stress responses, such as abscisic acid, drought, low-temperature response, and anaerobic response, were prevalent in the promoters of *VprAP3* and *VprPI* in *V*. *prionantha* (Table S25). Moreover, circadian-regulated *GI* is involved in signaling pathways for various abiotic stressors [62, 63]. In this scenario, energy was consumed to respond to the activation of abiotic and biotic stress genes; it would require less energy to trigger the expression of genes related to CH flower development, which is an energy-consuming process. Therefore, in response to LD, stress-response genes could be induced by VprGI, which might downregulate the expression of *VprAP3* and *VprPI* (Fig. 8). We also found that genes significantly co-expressed with *VprAP3* and *VprPI* were involved in glycolytic and fatty acid biosynthetic processes, and they were highly expressed in the floral buds induced under SDs, which might contribute to CH flower development (Fig. 8). Furthermore, the final and intermediate products of the glycolytic process and fatty acid biosynthetic process include the terpenoid, fatty acid, and phenolic compounds that are necessary for later flower development [67]. In addition, some genes related to cellulase involved in sugar–fatty acid processes, such as *GLC1* and *CEL1*, were expressed at high levels in CL flowers compared to CH flowers. The biochemical products of *GLC1* and *CEL1* encoding enzymes act as osmoprotectants, playing important roles in abiotic or biotic stress resistance [68–71]. Therefore, the photoperiod might regulate genes in the photoperiod pathway controlling flowering time, circadian clock-related genes, and floral organ identity genes without affecting floral organ initiation, and the involved transcription factors could further regulate genes involved in stress response and sugar–fatty acid metabolic processes, suggesting that their products serve as downstream molecules to determine whether there are CH flowers under SDs or CL flowers under LDs.

## Conclusions

The photoperiod drives or regulates the development and transition of CH–CL flowers in *V*. *prionantha*. SDs induced CH flower development, whereas LDs induced CL flowers. LDs induced earlier bloom and promoted the expression of the orthologous genes of *GI*, *FKF1/ADO3*, *APRR1*, *CO*, *FUL*, and *SOC1* in *V*. *prionantha*, whereas SDs promoted the expression of *VprUFO* and B-class MADS-box genes. Moreover, the expression of *VprAP3* and *VprPI* might relate to glycolytic, fatty acid biosynthetic, and sucrose metabolic processes and co-expression with *GAIP-B-like* and *YAB5*. *VprGI* and other genes in the photoperiod pathway related to the circadian clock were co-expressed with some stress-responsive genes but probably downregulated the expression of *VprAP3* and *VprPI* or *VprUFO*. Thus, we proposed an integrative model in which the transcription regulation of genes in the photoperiod pathway may coordinate the circadian clock, organ identity, and sugar–fatty acid/defense response for regulating the development of CH or CL in response to different photoperiods (Fig. 8), thus explaining the developmental evolution of dimorphic flowers.

## Methods

### Plant materials

Seeds of *V*. *prionantha* were collected and stored at the Northwest Normal University (Lanzhou, Gansu, China), and they are available upon request. The seedlings of *V*. *prionantha* were cultured according to a previous study [9]. The plants were grown at 22–28 °C under 10-, 12-, or 16-h daylight. To further study the effect of the photoperiod on CH and CL flower development, plants with CL flowers under 16-h daylight were switched to 10-h daylight to continue culture, and plants with CH flowers under 10-h daylight were switched to 16-h daylight to continue culture. The temperature was maintained by air conditioning. The humidity was maintained at around 48%, and the light intensity was 116 μmol m^−2^ s^−1^. Plants under different daylight conditions were grown in parallel on different shelves. The daylight length was controlled by a time controller on each shelf.

### Phenotypic analyses

The procedure of phenotypic analyses is similar to a previous description [9] without special note. Days to flower opening were counted from the beginning of every treatment under different photoperiods. The morphology of the flower buds was observed under a stereomicroscope (Olympus SZ61, Tokyo, Japan). The ratios of CH, inCL, and CL flowers were evaluated under 10-, 12-, and 16-h daylight. One hundred plants were observed in each photoperiod, and approximately 200 flower buds were counted in each case. In addition, after plants with CL flowers under 16-h daylight or CH flowers under 10-h daylight were switched to 10-h daylight or 16-h daylight, respectively, approximately 20 flower buds were observed under a stereomicroscope (Olympus SZ61, Tokyo, Japan) every 2, 4, 6, 8, and 10 days. The images were captured using a camera linked to a stereomicroscope (Olympus SZ61, Tokyo, Japan).

### Iso-seq data analysis

The total RNA from the indicated tissues of *V*. *prionantha* was extracted using an Rneasy Plant Mini Kit (Qiagen, Dusseldorf, Germany), and was qualified and quantified using a Nano Drop and Agilent 2100 bioanalyzer (Thermo Fisher Scientific, Waltham, Massachusetts, USA). We sequenced two libraries of full-length transcripts using PacBio Iso-Seq (BGI, Shenzhen, China). Two samples from the whole plants with CH flowers and the whole plants with CL flowers of *V*. *prionantha* (including the roots, stems, leaves, and floral buds) were collected. The RNA extracted from the samples was mixed in equal amounts to obtain transcriptomes from various plant tissues and treatments. Approximately 20 µg of total RNA (400 ng/mL) was used to prepare the cDNA library. First strand cDNA was synthesized by Clontech SMARTer PCR cDNA Synthesis Kit (Clontech, Mountain View, California, USA). The CDS Primer IIA was first annealed to the polyA^+^ tail of transcripts, followed by first-strand synthesis with SMARTScribe™ Reverse Transcriptase (Clontech, Mountain View, California, USA). The raw reads were processed using SMRT Link 5.0 software (https://www.pacb.com/support/software-downloads/). We clustered and polished the full-length (FL) sequences using an isoform-level clustering algorithm, iterative clustering for error correction (ICE), and the Quiver tool in SMRT Link software. The FL reads were further corrected with RNA-seq reads using LoRDEC [72] with the parameters of ‘-k19 -s 3 -T 4’, and redundancy was removed using Cd-hit [73] with the parameters of ‘-c 0.99 -T 10 -G 0 -aL 0 -aS 0.99 -AS 30 -d 0 -p 1’.

### RNA-seq data analysis

The total RNA was separately extracted and processed from three different development stages of floral buds, including the first development stage (S1), the third development stage (S3), and the fifth development stage (S5) of CH and CL flowers in *V*. *prionantha* using an Rneasy Plant Mini Kit (Qiagen, Dusseldorf, Germany). The polyA mRNAs were enriched using the magnetic oligo (dT) beads (NEB, Ipswich, Massachusetts, USA) and then were fragmented into 200 bp pieces. Next, random hexamer (N6) primers (Sangon Biotech, Shanghai, China) were used to build double strand cDNA libraries for all the samples. The cDNA libraries were constructed and sequenced using a BGISEQ-500 platform instrument (BGI, Shenzhen, China). The sequencing reads containing low-quality, adaptor-polluted, and high content of unknown bases (N) were removed before downstream analyses using Trimmomatic (V0.36) [74] with the parameters of ‘SE -phred33 ILLUMINACLIP: Trimmomatic-0.36/adapters/TruSeq3-SE. fa: 2:30:10 LEADING: 20 TRAILING: 20 SLIDINGWINDOW: 4:20 MINLEN: 50’.

### Transcript functional annotation

Blast, Blast2GO, and InterProScan were used to perform functional annotation with transcripts. Unigenes were searched against five databases, including Cluster of Orthologous Groups of proteins (COG), SwissProt, NCBI non-redundant (NR), Gene Ontology (GO), and Kyoto Encyclopedia of Genes and Genomes (KEGG) (www.kegg.jp/kegg/kegg1.html). Functional annotation of unigenes was obtained from sequence similarity alignment using the BLAST algorithm with a criterion of E-value < 1e-10.

### Gene family expansion and contraction analysis

To perform the gene family analysis, the amino acid sequences of *V*. *prionantha* and the relevant plant species (download from the NCBI database) were selected. We used OrthoFinder v2.4.1 [75] to perform gene family clustering with the markov clustering (MCL) inflation parameter of 1.5. We constructed a phylogenetic tree for *V*. *prionantha* and the selected plants based on single-copy orthologous genes. Amino acid sequences were extracted from the sequences of each single-copy gene family and concatenated into a supergene for each species. Multiple sequence alignment was performed with MUSCLE v3.8.31 [76], followed by the construction of a phylogenetic species tree using maximum likelihood in RAxML v8.2.12 [77]. Gene families were further filtered if one species had more than 200 genes. The remaining gene families were used to run CAFÉ (version4.1) [78] with default parameters.

### Gene mapping and expression analysis

Initially, RNA-seq clean reads from 18 samples were mapped to the unique non-redundant isoforms obtained from the ISO-seq data and used as the “reference transcripts” because no reference genome was available for *V*. *prionantha*. We mapped all clean-read transcripts using HISAT (V2.1.0) [79] with the parameters of ‘–dta -p 10 -x -U’. Any unmapped, qc-failed and PCR duplicated reads were filtered out, and only the alignments with high-quality (MAPQ >  = 10) were used for the downstream analysis. Based on the HISAT results, we calculated the gene expression level for each sample using StringTie (V1.3.3b) [80]. The normalized expression levels of the resultant isoforms were estimated based on transcripts per million (TPM) as the expression of each transcript for downstream analysis. Then, we calculated Pearson’s correlation between all samples, performed hierarchical clustering between all samples using hclust, performed PCA analysis with all samples using princomp, and drew the diagrams with the ggplot2 function in R (https://sourceforge.net/projects/ggplot2.mirror/).

### Detection of DEGs

The gene expression levels among the samples were examined based on RNA-seq data using the DEseq2 package [81] with default parameters. The high-quality total alignments were used in DEG detection. A *P*-adjust value < 0.05, and Log2 (fold change of Sample1/Sample2) for a gene > 1 or <  − 1 were used as the screening cutoffs for determining extremely significant differential gene expression between paired samples.

### Function enrichment analysis

The function of all genes collected was determined to elucidate the biological processes and pathways characterized in each developmental stage with R package clusterProfiler (v3.14) [82]. We obtained the results of biological process (BP), cellular component (CC), and molecular function (MF). The reference background set was the GO terms of all genes, and a *P*-value < 0.01 was regarded as significant.

### Identification and phylogenetic analysis of MADS-box genes

To identify MADS-box genes in *V*. *prionantha*, we firstly retrieved well-studied and annotated subtypes of MADS-box genes in *A*. *thaliana*, and then performed BLASTP analysis using these well-annotated *A*. *thaliana* MIKC-type MADS-box genes as queries, against the amino acid sequences in *V*. *prionantha* employing BLASTP (E-value < 10–5, and Identity score > 75). The identified putative MADS-box genes were further manually inspected using InterProScan (http://www.ebi.ac.uk/interpro/). To construct the phylogeny of these MADS-box genes, amino acid sequences were first aligned using MUSCLE v3.8.31 [76]. The poorly aligned regions were removed using trimAL v3117 [83] with a parameter of ‘-gt 0.9 -phylip’. A phylogenetic tree was constructed using maximum likelihood in RAxML v8.2.12 [77] under the “GTRGAMMA” model with 100 bootstrap replicates.

### Gene co-expression network analysis

We chose all DEGs to analyze gene co-expression network using the R package WGCNA (Weighted Correlation Network Analysis) Version: V1.48, and exactly followed the mannual protocol of this package [84]. It is briefly described as follows. The power parameter was pre-calculated by the pickSoft Threshold function. It can provide the appropriate soft-thresholding power for network construction by calculating the scale-free topology fit index for several powers. Turning adjacency into topological overlap was carried out, which measured the network connectivity of a gene, and defined as the sum of its adjacency with all other genes for network generation. The hierarchical clustering function was used to classify genes with similar expression profiles into modules based on topological overlap matrix (TOM) dissimilarity, with a minimum size of 30 and a maximum size of 5000 for the gene dendrogram. The dissimilarity of MEs was calculated to choose a cutline to merge some modules.

### Identification of significant modules and hub genes

Following the WGCNA pipeline [84], we calculated the correlation between MEs and traits (CH or CL flower) to identify the relevant modules. Next, gene significance (GS) was defined as the log10 transformation of the *P*-value in the linear regression between gene expression and trait information. In addition, module significance (MS) was defined as the average GS for all genes in a module. Each resulting model was visualized with Cytoscape software (http://www.cytoscape.org/). Key modules were selected and visualized using Cytoscape, and hub genes in networks were screened out using the Maximal Clique Centrality (MCC) score with the cytoHubba tool in Cytoscape (https://apps.cytoscape.org/apps/cytohubba) [85]. Hub genes ranked in the top 20 module networks were chosen as candidates for further analysis and validation.

### qRT-PCR analyses

The procedure for qRT-PCR was described in a previous study [9]. Briefly, gene expression was investigated using qRT-PCR in a Light-cycler480 (Roche, Mannheim, Germany) with an SYBR Premix Ex Taq Kit (Takara, Dalian, China) using gene-specific primers (Table S26). First-strand cDNA was synthesized from 2.0 μg of total RNA with oligo(dT) and M-MLV Reverse Transcriptase (Takara, Dalian, China). The expression of the endogenous 18S ribosomal RNA gene was used as an internal control [23]. The amplification conditions were initial denaturation at 95 °C for 30 s, followed by 40 cycles of 95 °C for 5 s, and 60 °C for 30 s. Three independent biological samples were used. The relative expression level was evaluated according to a previous description [86].

### Cloning and prediction of *VprAP3* and *VprPI* promoters

Based on the CDS sequences of *VprAP3* and *VprPI*, gDNA sequences were obtained using *LA Taq* (Takara, Dalian, China) with gene-specific primers (Table S26). According to the gDNA sequences, the promoter sequences were acquired using the Genome Walking Kit (Takara, Dalian, China). *Cis*-element prediction was analyzed by PlantCARE (http://bioinformatics.psb.ugent.be/webtools/plantcare/html/) and New PLACE (https://www.dna.affrc.go.jp/PLACE/?action=newplace).

### Statistical analyses

The mean ± standard deviation was calculated from at least three independent replicates. Significance differences were analyzed by Duncan in *SPASS13*.*0*.

## Supplementary Information

**Additional file 1: Supplementary Figures. **(Figures S1–S11 in one PDF file)

**Additional file 2: Supplementary Tables. **(Tables S1–S26 in one Excel file)

## Data Availability

All relevant supporting data can be found within the additional files accompanying this article. The promoter sequences of *VprPI* and *VprAP3* reported in this article can be found in NCBI (http://www.ncbi.nlm.nih.gov) under the accession numbers ON125105 and ON125106. The raw RNA-seq data have been deposited in the NGDC in this article can be found in BIG Data Center (https://ngdc.cncb.ac.cn/) as a BioProject PRJCA008547.

## Abbreviations

CH: Chasmogamous
CL: Cleistogamous
DEGs: Differentially expressed genes
ORF: Open reading frame
CDS: Coding sequence
cDNA: Complementary DNA;
qRT-PCR: Real-time quantitative reverse transcription PCR
PCA: Principal component analysis
GO: Gene ontology
KEGG: Kyoto encyclopedia of genes and genomes
WGCNA: Weighted correlation network analysis
