# Supplementary material for: Transcriptomic comparison sheds new light on regulatory networks for dimorphic flower development in response to photoperiod in Viola prionantha
Source: BMC Plant Biol. 2022 Jul 12;22:336. doi: 10.1186/s12870-022-03732-4 (PMC9277944; doi:10.1186/s12870-022-03732-4)
Supplement: Supplementary file 1 — Additional file 1: Supplementary Figures. (Figures S1–S11 in one PDF file) [file 12870_2022_3732_MOESM1_ESM.pdf]

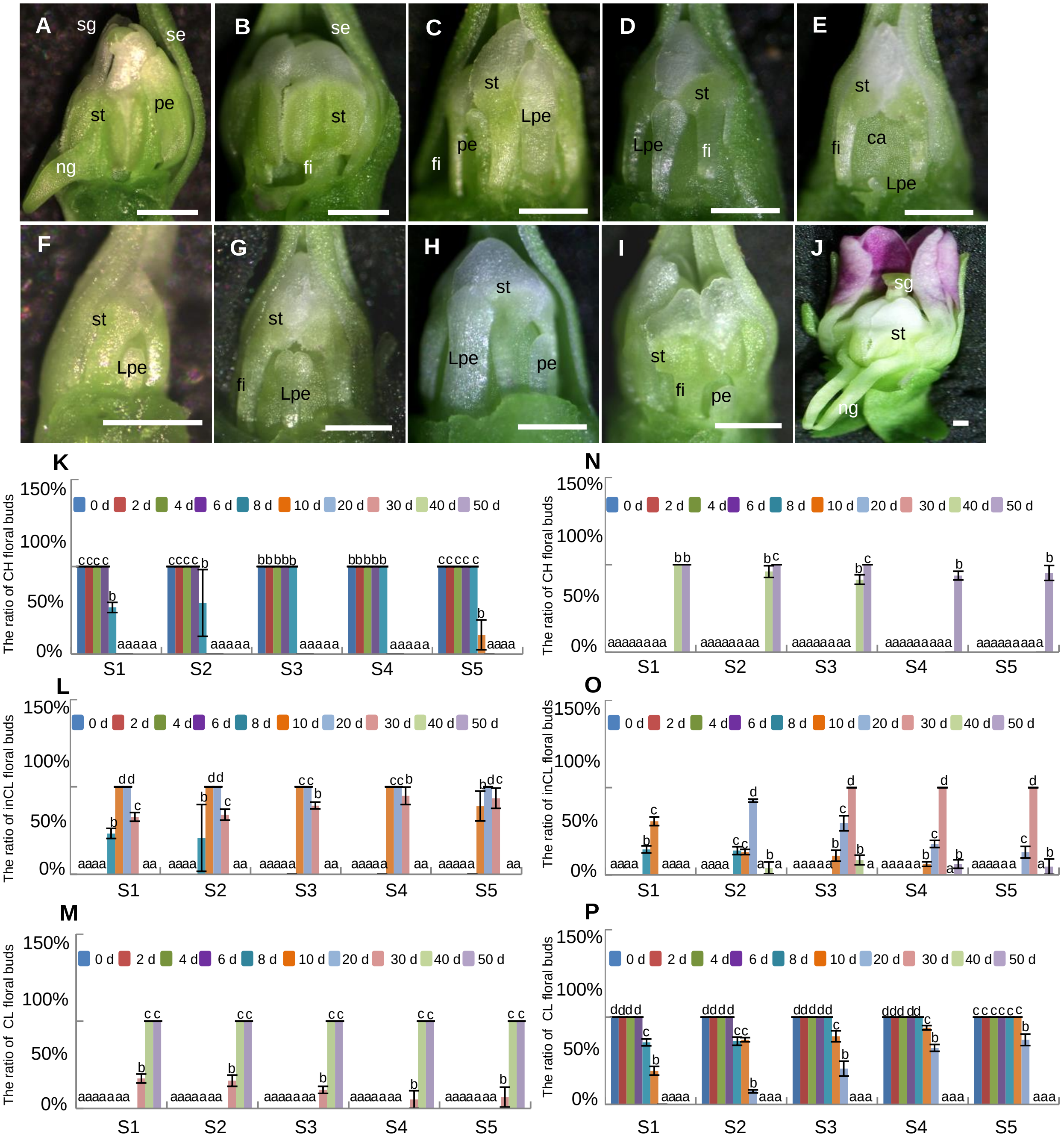

Figure S1 Floral morphological variations after *V. prionantha* plants were interconverted between photoperiods (**A**–**E**) Floral morphological variations after 8 (A), 10 (B), 20 (C), 30 (D), and 40/50 (E) days when the *V. prionantha* plants were switched from 10-h daylight to 16-h daylight. (**F–J**) Floral morphological variations after 8 (F), 10 (G), 20 (H), 30 (I), and 40/50 (J) days when the *V. prionantha* plants were switched from 16-h daylight to 10-h daylight. Bars = 500 um in A–J. se, sepal; pe, petal; Lpe, lower petal; st, stamen; ca, carpel; sg, stigma; an, anther; sc, stamen cap; fi, filament; ng, nectar gland. (**K**–**M**) The statistics of floral morphological variations after the *V. prionantha* plants were switched from 10-h daylight to 16-h daylight. (**N**–**P**) The statistics of floral morphological variations after the *V. prionantha* plants were switched from 16-h daylight to 10-h daylight. S1–S5, defined in Figure 1. Standard errors are provided, and lowercase letters (a, b, c, and d) indicate significant differences ($P < 0.05$).

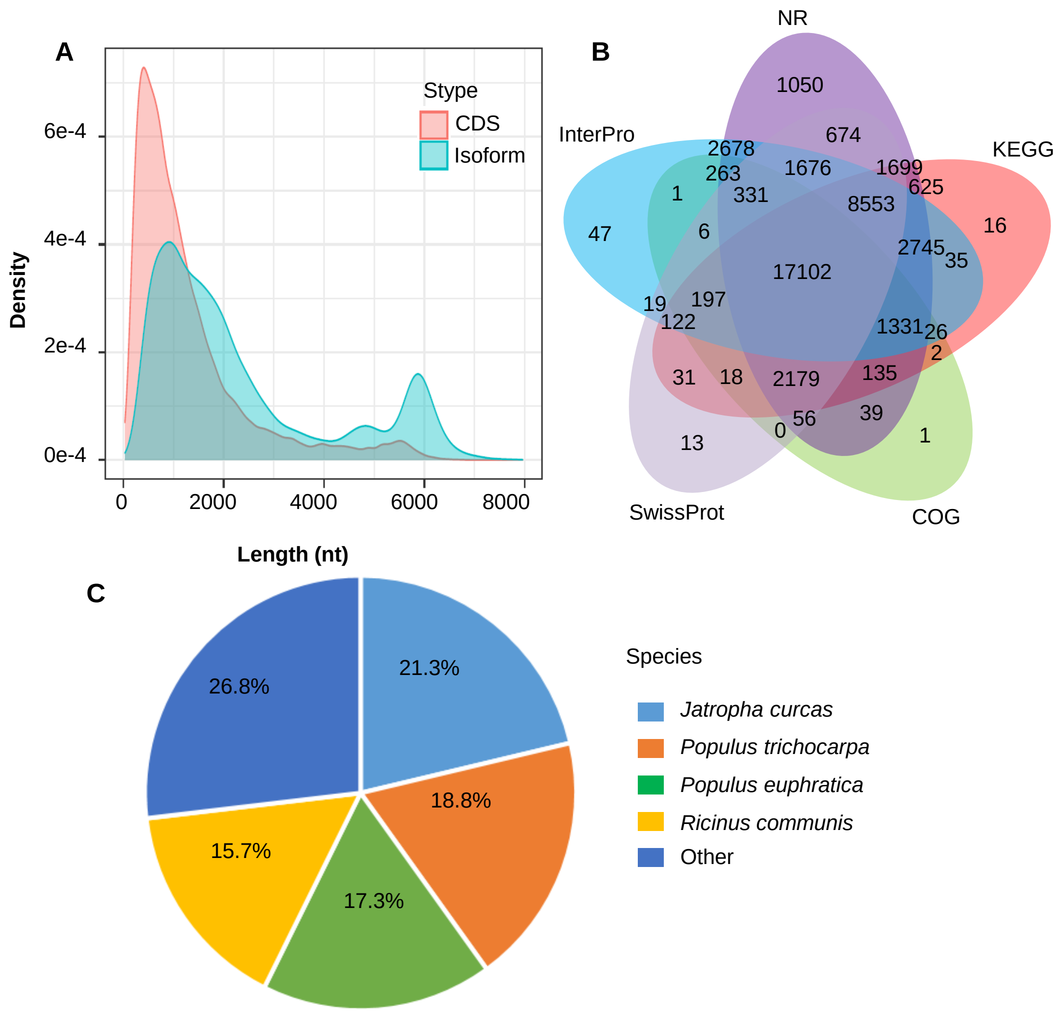

Figure S2 Full-length transcriptome analysis

(**A**) Density plot for the length distribution of final consensus isoforms and CDS. (**B**) The number of transcripts which were annotated by multiple databases. Venn diagram displays the overlaps between NR, COG, KEGG (www.kegg.jp/kegg/kegg1.html), Swissprot, and Interpro. **(C)** Distribution of hit plant species from BLAST search of PacBio-isoforms pie chart shows the fraction of hit plant species based on the best hit obtained from BLASTX search of PacBio transcripts against NR database.

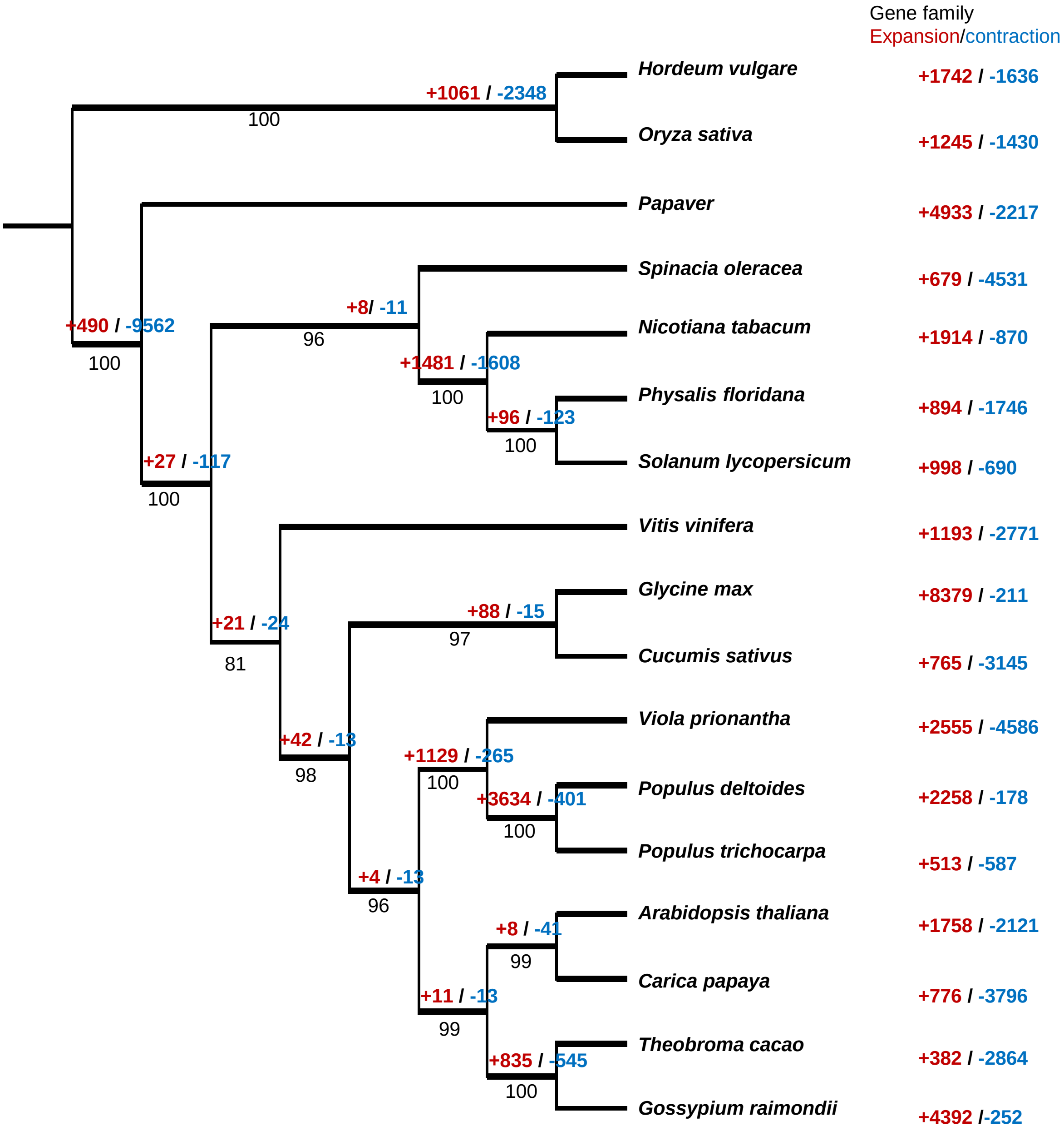

Figure S3 Expansion and contraction of gene families in *V. prionantha*
The phylogenetic tree of 17 plant species was constructed based on single-copy orthologous genes using maximum likehood method with Raxml. Bootstrap support numbers are given at bottom. Red numbers indicate the number of gene families expanded in a species relative to the closest species during evolution, and blue numbers indicate the number of gene families contracted.

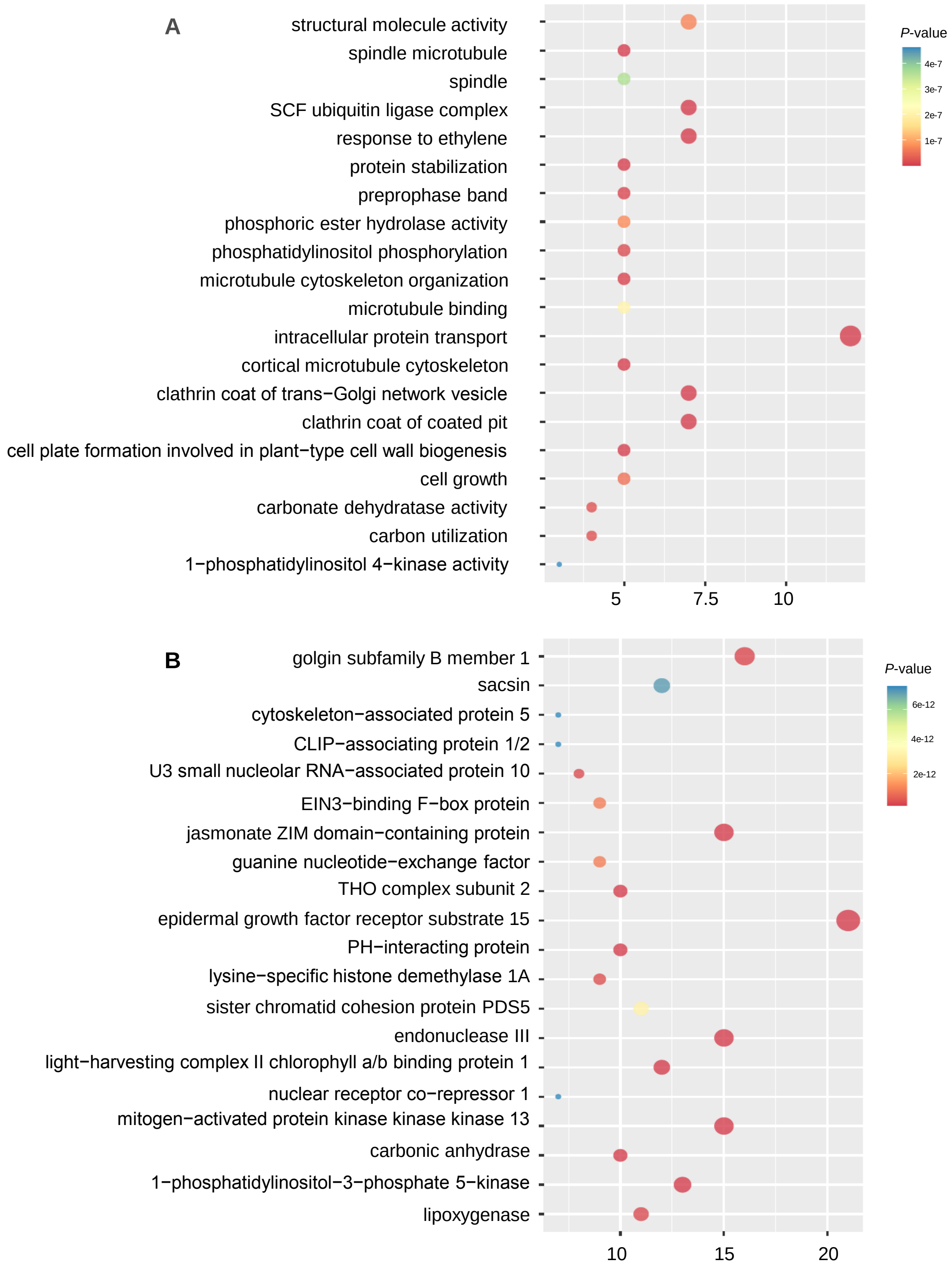

Figure S4 Significantly expanded gene families in *V. prionantha*
(**A**) GO enrichment terms. (**B**) KEGG enrichment terms (www.kegg.jp/kegg/kegg1.html). The top 20 terms significantly enriched are shown.

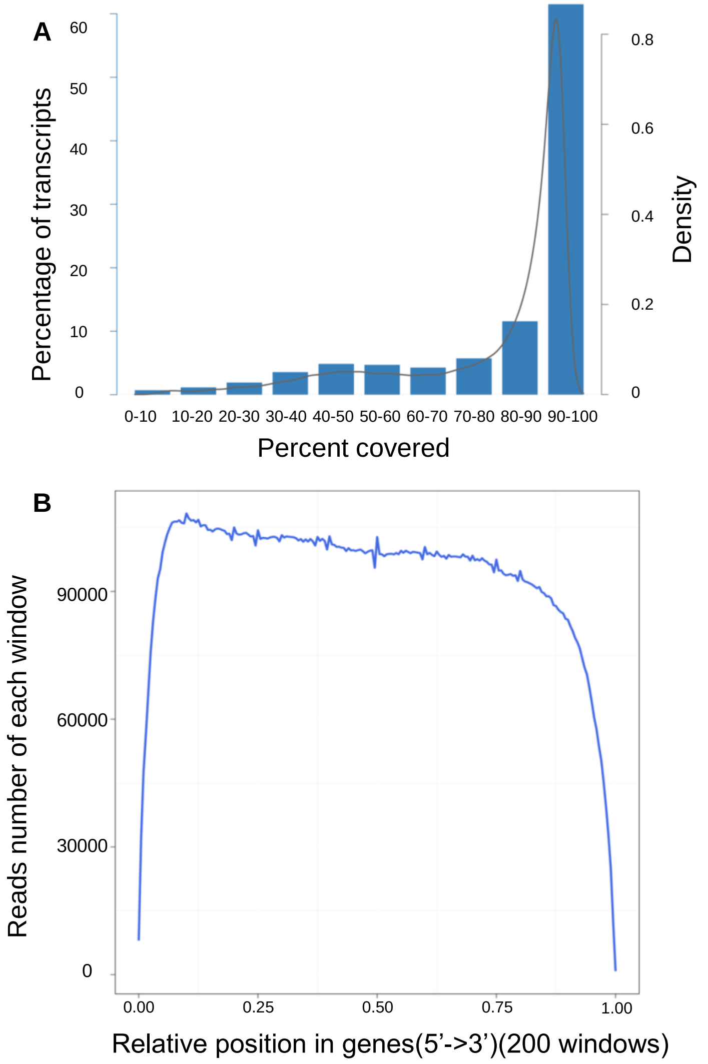

Figure S5 RNA-seq read coverage and distribution on the full-length transcripts
(**A**) RNA-seq read coverage. X axis represents the coverage ratio of each transcript. Y axis on left side represents the percentage of total transcripts. Y axis on right side represents the density of total transcripts. (**B**) RNA-seq read distribution. X axis represents the position along transcripts. Y axis represents the number of reads.

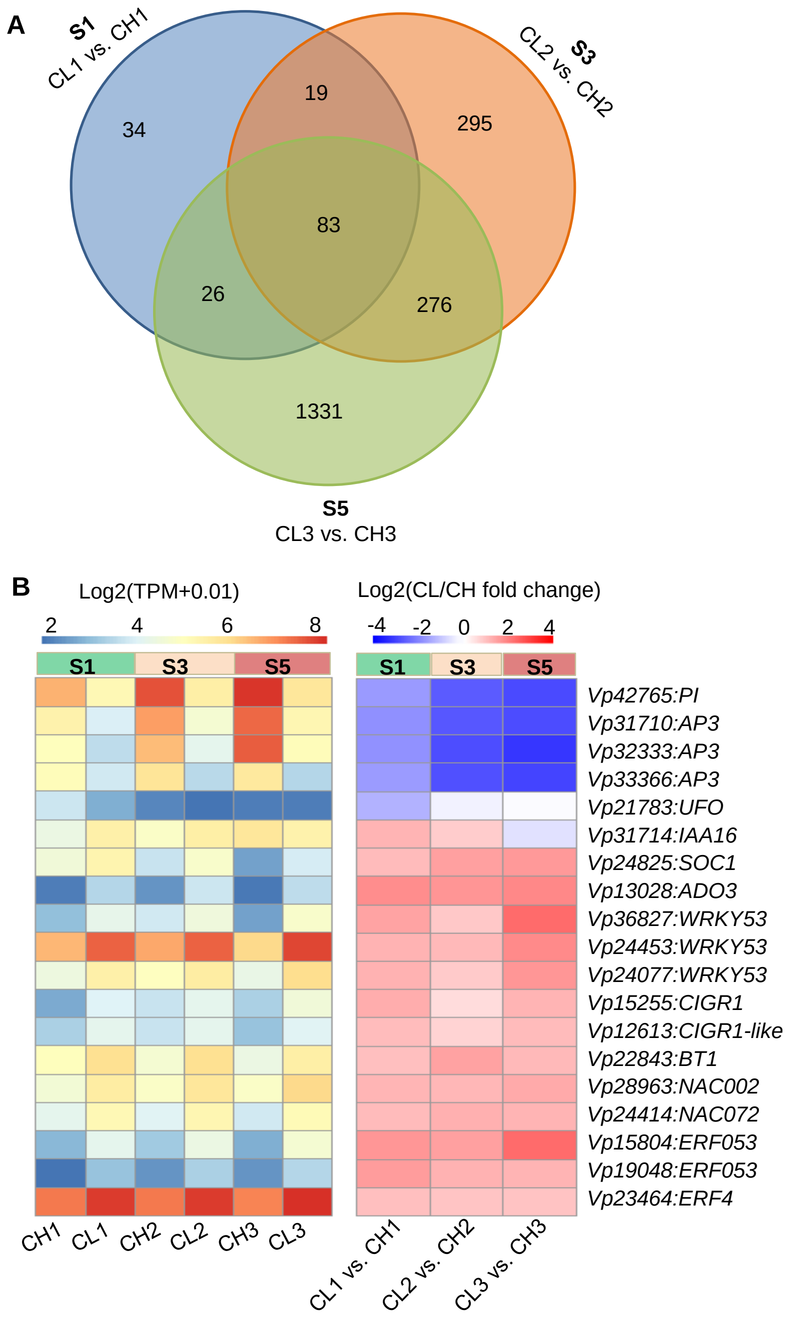

Figure S6 Statistics and expression profile of the DEGs for three stages of *V. prionantha*
(A) Venn diagram of the number of DEGs (with a *p-adj* < 0.05 and an expression level change of at least 1-fold, |log2FC| >1) for the indicated three stages. **(B)** Expression profile of the DEGs involved in the transcription regulation. Heatmap displays the expression TPM (left) and TPM fold change (CL/CH) (right) in each flowering stage of *V. prionantha.* Red represents gene high-expression in CL and blue represents low-expression in CL. S1, S3 and S5 are respectively floral buds of the first, third, and fifth development stages defined in Figure 1.

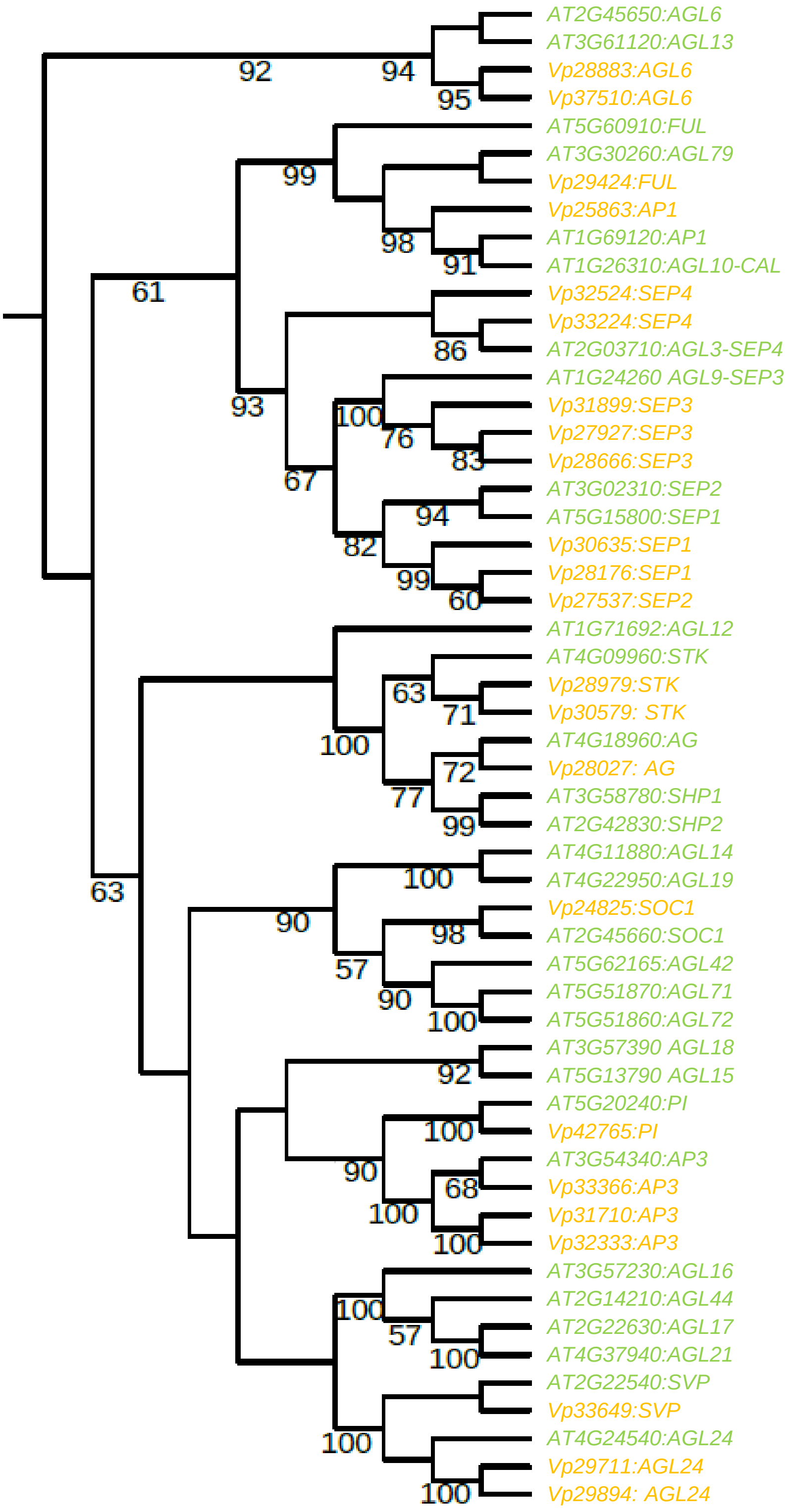

Figure S7 The phylogeny of MIKC-type MADS-box genes

MADS-box genes of *A. thaliana* and *V. prionantha* are respectively marked by green and orange. The tree was constructed using maximum likelihood in Raxml. Bootstrap values > 50% are shown.

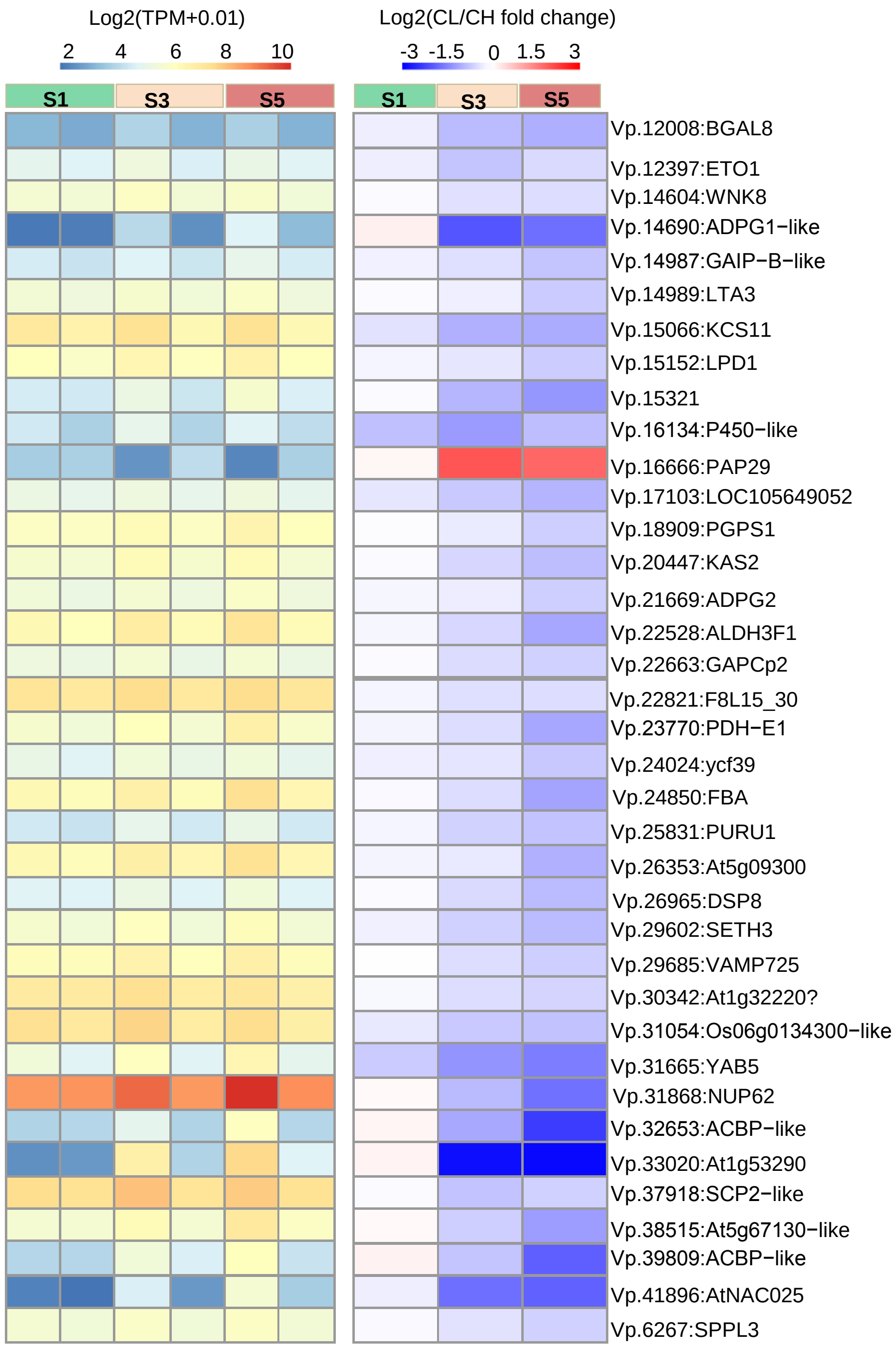

Figure S8 Expression profile of the DEGs co-expressed with *AP3* and *PI*
Heatmap displays the expression TPM (left) and TPM fold change (CL/CH) (right) in each flowering stage of *V. prionantha*. Red represents gene high-expression in CL and blue represents low-expression in CL. S1, S3 and S5 are respectively floral buds of the first, third, and fifth development stages defined in Figure 1.

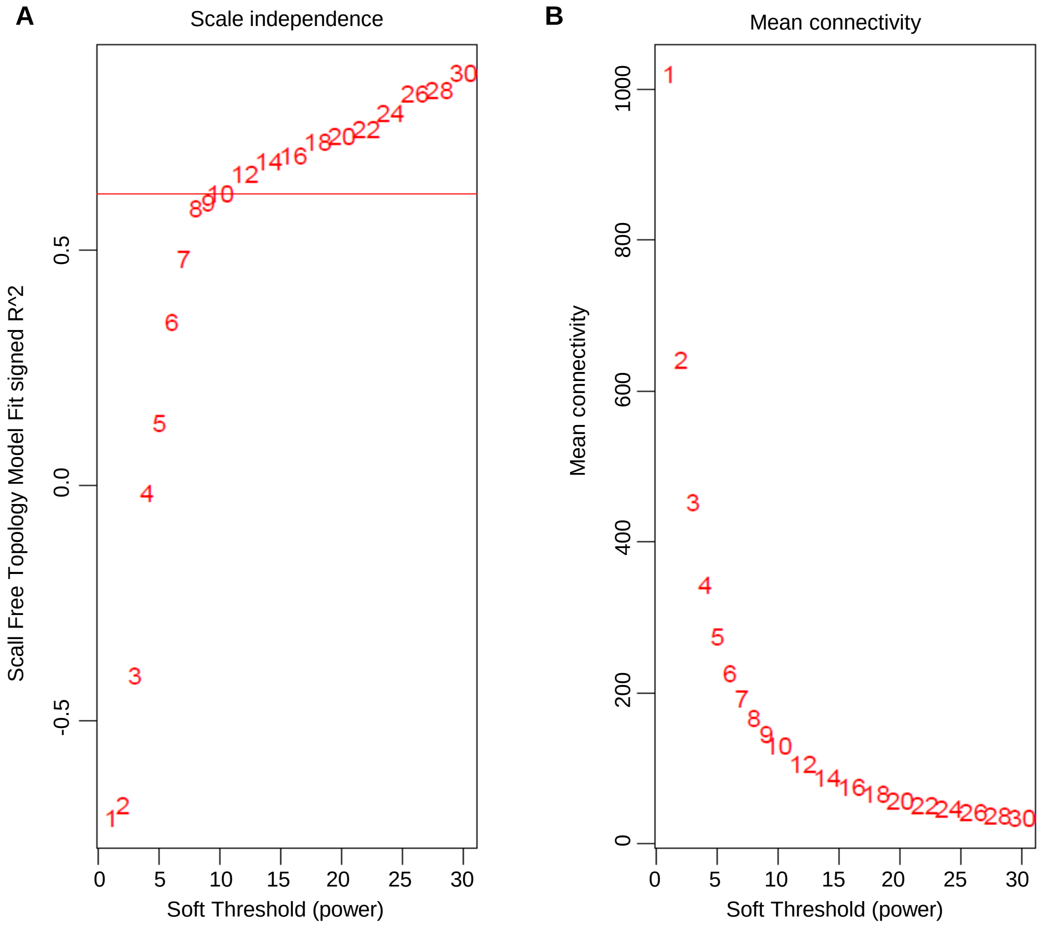

Figure S9 Determination of soft-thresholding power in WGCNA
(**A**) Analysis of the scale-free fit index for various soft-thresholding powers (β). (**B**) Analysis of the mean connectivity for various soft-thresholding powers.

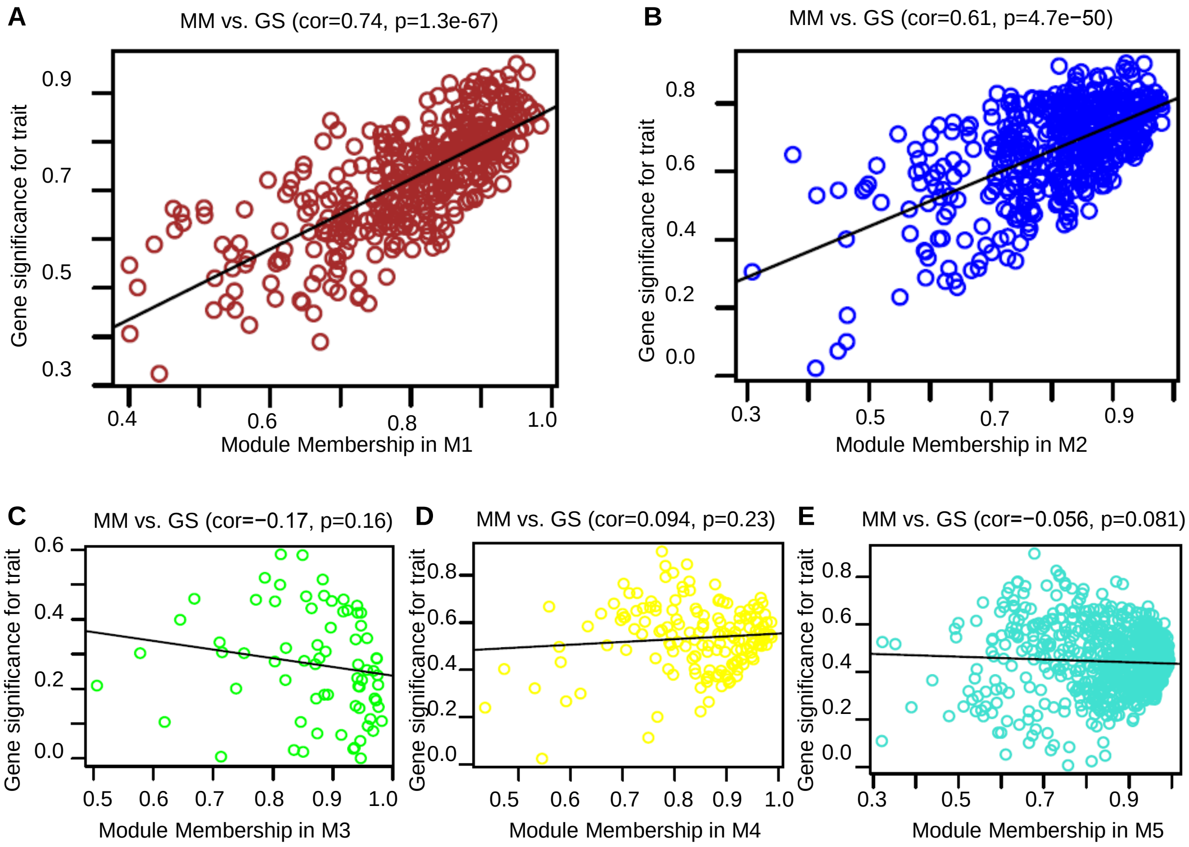

Figure S10 Scatterplot of gene significance (GS) vs module membership (MM) in different modules (**A–E**) The relationship of GS and MM in each module (from M1 to M5). GS and MM are highly correlated in M1 and M2 modules, illustrating that genes highly associated with the CH-CL flower development are the most central elements in the two modules.

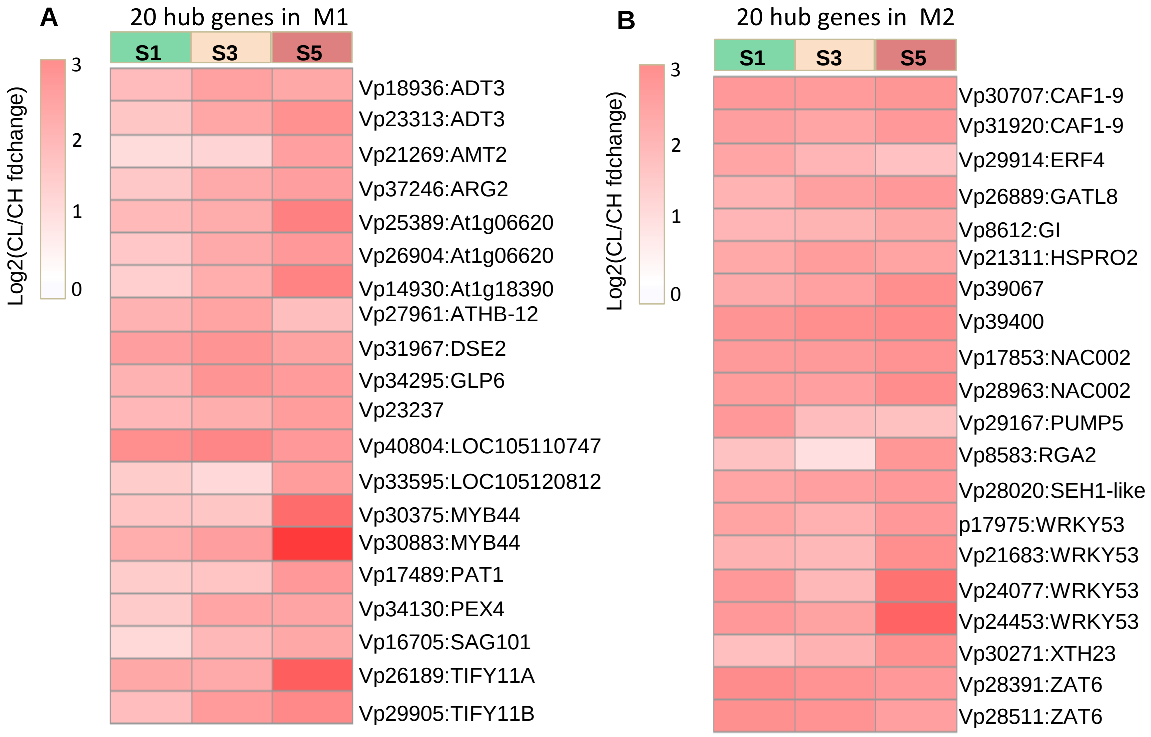

Figure S11 Expression profile of genes in M1 and M2
(**A**) Expression profile of the top 20 genes in M1 ranked by MCC. (**B**) Expression profile of the top 20 genes in M2 ranked by MCC. Heatmap displays the expression TPM (left) and TPM fold change (CL/CH) (right) in each flowering stage of *V. prionantha.* Red represents gene high-expression in CL. S1, S3, and S5 are respectively floral buds of the first, third, and fifth development stages defined in Figure 1.
